# Global genetic diversity, lineage distribution, and *Wolbachia* infection of the alfalfa weevil *Hypera postica* (Coleoptera: Curculionidae)

**DOI:** 10.1002/ece3.5474

**Published:** 2019-08-06

**Authors:** Ehsan Sanaei, Martin Husemann, Marjan Seiedy, Michael Rethwisch, Midori Tuda, Teodora B. Toshova, Min Jee Kim, Daniela Atanasova, Iksoo Kim

**Affiliations:** ^1^ Department of Applied Biology College of Agriculture and Life Science Chonnam National University Gwnagju Korea; ^2^ School of Biological Science University of Queensland Brisbane Queensland Australia; ^3^ Centrum für Naturkunde Universität Hamburg Hamburg Germany; ^4^ School of Biology and Center of Excellence in Phylogeny of Living Organisms College of Science University of Tehran Tehran Iran; ^5^ University of California Cooperative Extension Blythe CA USA; ^6^ Faculty of Agriculture Institute of Biological Control Kyushu University Fukuoka Japan; ^7^ Laboratory of Insect Natural Enemies Department of Bioresource Sciences Faculty of Agriculture Kyushu University Fukuoka Japan; ^8^ Institute of Biodiversity and Ecosystem Research Bulgarian Academy of Sciences Sofia Bulgaria; ^9^ Department of Entomology Faculty of Plant Protection and Agroecology Agricultural University Plovdiv Bulgaria

**Keywords:** biogeography, genetic diversity, Hypera postica, invasive population, mitochondrial lineage, Wolbachia

## Abstract

The alfalfa weevil (*Hypera postica*) is a well‐known example of a worldwide‐distributed pest with high genetic variation. Based on the mitochondrial genes, the alfalfa weevil clusters into two main mitochondrial lineages. However, there is no clear picture of the global diversity and distribution of these lineages; neither the drivers of its diversification are known. However, it appears likely that historic demographic events including founder effects played a role. In addition, *Wolbachia*, a widespread intracellular parasite/symbiont, likely played an important role in the evolution of the species. *Wolbachia* infection so far was only detected in the Western lineage of *H. postica* with no information on the infecting strain, its frequency, and its consequences on the genetic diversity of the host. We here used a combination of mitochondrial and nuclear sequences of the host and sequence information on *Wolbachia* to document the distribution of strains and the degree of infection. The Eastern lineage has a higher genetic diversity and is found in the Mediterranean, the Middle East, Eastern Europe, and eastern America, whereas the less diverse Western lineage is found in Central Europe and the western America. Both lineages are infected with the same common strain of *Wolbachia* belonging to Supergroup B. Based on neutrality tests, selection tests, and the current distribution and diversification of *Wolbachia* in *H. postica*, we suggested the *Wolbachia* infection did not shape genetic diversity of the host. The introduced populations in the United States are generally genetically less diverse, which is in line with founder effects.

## 1 INTRODUCTION

The genetic diversity and composition of cosmopolitan pest species can be shaped by several forces (Bazin, Glémin, & Galtier, 2006) including mutations (Wright, 2001), human‐mediated or natural gene flow (Roman & Darling, 2007; Sanaei et al., 2016), and genetic drift (Gonzalez‐Quevedo, Spurgin, Illera, & Richardson, 2015; Grubaugh et al., 2016; Hershberg et al., 2008; Stuckas et al., 2017), including bottlenecks and founder effects (Balick, Do, Cassa, Reich, & Sunyaev, 2015; Hundertmark & Van Daele, 2010). However, especially in insects, some maternally inherited microorganism such as *Wolbachia* has the ability to manipulate the host's reproductive system and consequently alters the mitochondrial genetic patterns of the host population (Dewayne Shoemaker, Keller, & Ross, 2003; Henry, May, Acheampong, Gillespie, & Roitberg, 2010; Lajeunesse & Forbes, 2002). The alfalfa weevil, *Hypera postica* (Gyllenhal, 1813) (Coleoptera: Curculionidae), is a prime example of such an insect pest species with a complex genetic structure including coexistence of the two distinct mitochondrial lineages across the native and invasive distribution range (Iwase, Nakahira, Tuda, Kagoshima, & Takagi, 2015; Sanaei et al., 2016). Yet, so far, distribution patterns are only marginally understood and we have little information on intralineage divergence patterns and their causes. Understanding the patterns and drivers of genetic diversification may also aid in biological pest control as local diversified pest lineages may show different patterns of local adaptation and resistance patterns against controlling agents (Chen & Dorn, 2010; Denholm, Cahill, Dennehy, & Horowitz, 1998; Lucas, 2011).

The alfalfa weevil is a widespread common pest of alfalfa *Medicago sativa* Linnaeus, 1753 (Fabaceae) (Sanaei, Seiedy, & de Castro, 2015; Summers, 1998). A recent study suggested that this species has a Palearctic origin, but has undergone human‐mediated translocations to several parts of the world (Sanaei et al., 2016). The invasion of *H. postica* to much of the Holarctic region is well documented with the first introductions to the America in 1904 (Titus, 1909), Japan in 1982 (Kuwata, Tokuda, Yamaguchi, & Yukawa, 2005), and Korea in 2002 (Hong & Kim, 2002). Historically, the alfalfa weevil populations in the America were categorized into three lineages which were called Eastern, Egyptian, and Western strains (Bundy, Smith, English, Sutton, & Hanson, 2005). Each strain was introduced to America via an independent route and time (Radcliffe & Flanders, 1998). While there is a lack of distinct morphological characters (Bland, 1984; Pienkowski, Hsieh, & Lecato, 1969; Sanaei, Seiedy, & Momtazi, 2015b), several fluctuating ecological traits were diagnosed to be strain specific including response to parasitoids and location of pupation (Coles & Day, 1977; Dewitt & Armbrust, 1972; Litsinger & Apple, 1973). However, the usefulness of these characters is questionable as strains with certain ecological characters were determined only based on their location and most of the mentioned characters were not applicable or showed huge variation and overlap among strains (Bundy et al., 2005; Sanaei et al., 2016).

At the end of the 20th century, studies started using molecular markers to quantify strain divergence (Erney, Pruess, Danielson, & Powers, 1996; Hsaio, 1996). The results of mitochondrial analyses (parts of COI and CytB genes with a total length of 1,031 bp) showed that the Eastern and Egyptian strains were similar and together have an approximate nucleotide difference of 5% to the Western strain (Erney et al., 1996). While nuclear genes failed to recover any pattern of diversification (Böttger, Bundy, Oesterle, & Hanson, 2013; for more information on genes please refer to Appendices S1 and S2), several studies confirmed the strong mitochondrial gene divergence between Western and Eastern (including American Eastern and Egyptian strains) lineages (Böttger et al., 2013; Iwase, Nakahira, et al., 2015; Kuwata et al., 2005; Sanaei et al., 2016). However, the genetic diversity underlying each lineage remains unknown.

Several factors may have contributed to the mitochondrial lineage divergence and diversification in *H. postica*; these may include population history, adaptation to different hosts, or disrupted interpopulation gene flow (Iwase, Nakahira, et al., 2015; Iwase, Tani, et al., 2015; Sanaei & Seiedy, 2016; Sanaei, Seiedy, & Momtazi, 2015a; Sanaei et al., 2016). One additional potentially important agent is the intracellular alpha‐proteobacteria *Wolbachia* (Hertig, 1936; Werren, 1997). Several hundreds of strains of *Wolbachia* have been diagnosed so far within nematodes and arthropods with various effects on their hosts (Gerth & Bleidorn, 2017; Werren, Windsor, & Guo, 1995; Zug & Hammerstein, 2012). Recently, it has been estimated that *Wolbachia* infects 38.3% of all beetle species (Kajtoch & Kotásková, 2018). *Wolbachia* is transmitted to the host progeny via the cytoplasm of the eggs and is therefore maternally transferred to the next generation (Bourtzis, Dobson, Braig, & O'Neill, 1998; Branca, Vavre, Silvain, & Dupas, 2009; Hoffmann, 1988; Telschow, Hammerstein, & Werren, 2002). *Wolbachia* manipulates the reproductive biology of its host and can cause a broad range of induced phenotypes such as male killing (Fialho & Stevens, 2000; Hurst et al., 1999), feminization (Bouchon, Rigaud, & Juchault, 1998; Kageyama, Nishimura, Hoshizaki, & Ishikawa, 2002), parthenogenesis (Huigens & Stouthamer, 2003; Weeks & Breeuwer, 2001), and cytoplasmic incompatibility (CI) between infected males and uninfected females or between individuals infected with different strains (Hale & Hoffmann, 1990; LePage et al., 2017; Prakash & Puttaraju, 2007; Yen & Barr, 1971). *Wolbachia* has also been suggested to cause reproductive isolation between infected and uninfected populations; as such it may promote speciation (Kaiser et al., 2015; Lefoulon et al., 2016). Maternal transmission of *Wolbachia* in combination with *Wolbachia*‐induced CI can lead to the hitchhiking of an infected mitochondrial haplotype and thus may decrease mtDNA genetic diversity (Minard et al., 2015; Narita, Nomura, Kato, & Fukatsu, 2006; Pannebakker, Zwaan, Beukeboom, & Alphen, 2004). Therefore, infected and uninfected populations may follow different evolutionary trajectories, which can significantly modify a species gene pool even within a short time period (Baldo, Bordenstein, Wernegreen, & Werren, 2006; Bordenstein & Wernegreen, 2004; Correa & Ballard, 2016).

The American populations of the alfalfa weevil were one of the first hosts for which CI (Hsaio, 1996; Hsiao & Hsiao, 1985a, 1985b) and potential sex ratio disorder (Hsiao & Hsiao, 1985b) caused by *Wolbachia* were diagnosed. The unsuccessful cross mating between uninfected Eastern and infected Western lineage individuals indicated a reproduction barrier caused by bidirectional CI (Hsiao & Hsiao, 1985b). A study of American populations further suggested that the Western lineage is naturally infected, whereas the Eastern lineage is naturally resistant to *Wolbachia* (Bundy et al., 2005; Hsaio, 1996). However, in Japan, an Eastern lineage sample was found to be infected with *Wolbachia;* yet, here none of the examined Western lineage samples were infected (Iwase, Tani, et al., 2015). Recently, it has also been shown that in the absence of *Wolbachia* infection in Japan, both lineages produce viable eggs (Iwase & Tani, 2016) providing further evidence that CI induced by *Wolbachia* did not result in complete reproductive isolation and hence speciation. However, we still know little on the frequency, type, and diversity of *Wolbachia* in the worldwide populations of *H. postica*. Further, its effect on host genetic diversification remains unclear. While the genetic composition of invasive populations may be the result of historical demographic events (Estoup & Guillemaud, 2010; Szűcs, Melbourne, Tuff, Weiss‐Lehman, & Hufbauer, 2017), the potential role of *Wolbachia* in the reduction of genetic diversity of alfalfa weevil populations remains unknown.

In order to fill this knowledge gap, we generated a large molecular dataset (mitochondrial and nuclear DNA and molecular information on *Wolbachia*) of several *H. postica* populations from different parts of the world. For the first time, we mapped the current distribution of the two lineages of alfalfa weevil in its native and invasive distribution range covering North America, Europe, the Middle East, and Eastern Asia. We further report the degrees of intra‐and interpopulation divergence and conducted neutrality analyses to test for signs of selection potentially caused by *Wolbachia*. Finally, we report the degree of infection for all populations and discuss its potential role for the genetic diversification of the host species.

## 2 METHODS

### 2.1 Sampling

Specimens of *H. postica* were collected during several field trips from 2012 to 2016 (Table 1). Samples of various populations from a wide geographic range were collected mostly from alfalfa fields, but each Japanese and Korean populations were collected from California burclover (*Medicago polymorpha* L.) and Chinese milk vetch (*Astragalus sinicus* L.), respectively; Lozitsa (Bulgaria) samples were collected from Bird vetch (*Vicia cracca* L.). Samples were preserved in pure ethanol and stored in a freezer at −20°C until further processing.

**Table 1:** Population sample list with infection rate

Population name	Collectors	Elevation (m)	Date	Sample size	Infection rate
Jovein, Iran	E. Sanaei	1,140	27 April 2014	17	0
Karaj, Iran	E. Sanaei	1,315	26 April 2014	26	0
Hamedan, Iran	E. Sanaei	1,646	29 April 2014	6	0
Tuyserkan, Iran	E. Sanaei	1,657	29 April 2014	26	0
Taleghan, Iran	E. Sanaei	1,920	8 May 2015	6	0
Toscana, Italy	E. Sanaei	960	24 May 2015	3	0
Montana 1, USA	T. Rand	50	5 Jun 2014	32	25%
Montana 2, USA	T. Rand	0	10 Jun 2014	25	66%
Missouri, USA	B. Puttler	234	30 March 2016	18	0
Gwangju, Korea	E. Sanaei	33	30 April 2016	21	19%
Warsaw, Poland	M. A. Mazur	190	18 May 2012	1	100%
Chraberce, Czech Republic	J. Skuhrovec	338	14 May 2014	4	75%
Nebraska, USA	M. D. Rethwisch	490	6 May 2016	28	7%
California, USA	L. Godfrey	171	10 May 2016	23	27%
Japan, Okinawa	M. Tuda	50	4 April 2015	20	55%
Plovdiv, Bulgaria	D. Atanasova	157	5 May 2016	24	25%
Knezha, Bulgaria	T. B. Toshova	121	23 April 2016	16	6%
Lozitsa, Bulgaria	T. B. Toshova	179	3 Jun 2016	1	100%

For this study, we used a total of 292 specimens of *H. postica*. Further, one specimen of *Hypera mele* (Fabricius, 1792) (Iran, Taleghan), three specimens of *Hypera viciae* (Gyllenhal, 1813) (Bulgaria, Lozitsa), and one specimen of *Brachypera zoilus* (Scopoli, 1763) (Bulgaria, Knezha) (Appendix S1) were included as out‐groups. These were also tested for *Wolbachia* to investigate whether closely related species are infected with the same *Wolbachia* strains.

### 2.2 Molecular analyses

DNA was extracted from hind legs using the Wizard™ Genomic DNA Extraction Kit (Promega). Two mitochondrial (COI and CytB) and two nuclear genes (EF1a and CAD) were amplified with specifically designed primers (for information on amplicon size, primer design, detailed PCR, and sequencing conditions, please refer to Appendix S2). Sequences were checked and aligned using ChromasPro v. 1.7.7 (http://technelysium.com.au/wp/chromaspro/) and ClustalX (Thompson, Higgins, & Gibson, 1997).

### 2.3 Basic statistics and tests for selection

Estimates of genetic diversity the number of segregating sites (S), number of haplotypes (H), haplotype diversity (Hd), the average number of nucleotide differences (K), nucleotide diversity (pi), singleton variable sites (V), and parsimony informative sites (P) were assessed using DnaSP v. 4.0 (Swofford, 2002) and Arlequin v. 3.5 (Excoffier & Lischer, 2010) at the population and lineage levels. To infer genetic diversity, we used the two mitochondrial and the nuclear genes separately. Pairwise Φ_ST_ values (Hudson, Slatkin, & Maddison, 1992) were calculated in DnaSP. In order to quantify population differences, an analysis of molecular variance (AMOVA; Excoffier, Smouse, & Quattro, 1992) was performed based on the mitochondrial genes in Arlequin. Six geographic groups were defined for comparative analysis: (a) Iranian samples, except the Western lineage sample of Taleghan, (b) all Eastern lineage samples from Bulgaria, (c) all American Western populations, (d) American Eastern population (Missouri population), (e) the Eastern lineage samples from Japan and Korea, and (f) the Western lineage samples from Japan and Korea. According to the low number of samples from Italian, Czech and Polish populations and few Western lineage samples from Iran and Bulgaria, we excluded these from AMOVA. To assess whether the examined genes evolved randomly or not, Tajima's D (Tajima, 1989), Fu's D, and Li's D test (Fu & Li, 1993) were performed in DnaSP. Based on the flight ability (Prokopy & Gyrisco, 1965) and human‐mediated translocations of *H. postica* (Sanaei et al., 2016), we assumed our populations as panmictic (nonstructured). In the absence of direct selection (null hypothesis), negative significant values indicate a recent sweep or bottleneck, whereas positive values point to balancing selection during a possible bottleneck (Mayer et al., 2015). A similar neutrality test was conducted for CAD sequences. Hudson, Kreitman, and Montserrat (HKA; Hudson, Kreitman, & Aguadé, 1987) and McDonald–Kreitman tests (McDonald & Kreitman, 1991) were used to test for selective pressure on mitochondrial genes, which may be caused by *Wolbachia* (positive test for mitochondrial genome and negative for nuclear) or by demography events (positive test for both nuclear and mitochondrial gene). Three groups were defined for these tests: (a) Western lineage versus Eastern lineage (b) Eastern endemic infected (Bulgaria) versus Eastern endemic uninfected (Iran, Italy), (c) Eastern infected (Bulgaria) versus Eastern uninfected (Iran, Italy, Missouri).

The ratio of nonsynonymous to synonymous substitutions (dN/dS) for Eastern and Western lineages observed on the phylogenetic tree was measured with CodeML implemented in the PAML package (Yang, 1997) of PamlX (Xu & Yang, 2013) following the guidelines to test for natural selection effects on a protein‐coding mitochondrial gene (Jeffares, Tomiczek, Sojo, & Reis, 2015). Therefore, in order to estimate the dN/dS ratio for all branches, COI and CytB sequences and the maximum‐likelihood phylogenetic trees constructed by RAxML v. 8 (Stamatakis, 2014) were used.

### 2.4 Phylogenetic analyses

To reconstruct a phylogenetic tree for the sampled alfalfa weevil populations, we used MrBayes v. 3.2.1 (Ronquist & Huelsenbeck, 2003). In a first step, the best substitution model was estimated using jModelTest v. 2.1.10 (Darriba, Taboada, Doallo, & Posada, 2012). All four loci were concatenated using SequenceMatrix v. 1.8 (Vaidya, Lohman, & Meier, 2011). PAUP was used to perform a partition‐homogeneity test with 10,000 replicates, which suggested congruence of all partitions (*p* value >.05). We ran MrBayes for 10 million generations, sampling every 1,000 generations yielding a total of 10,000 trees; the first 25% of trees were discarded as burn‐in. Convergence was checked using the average standard deviations of split frequencies, which were below 0.005 and effective sample sizes (above 200). Trees were visualized and edited with FigTree v. 1.4.2 (Rambaut, 2006) and TreeGraph v. 2 (Stöver & Müller, 2010).

Using the mtDNA data, a TCS haplotype network (Clement, Snell, Walker, Posada, & Crandall, 2002) was generated and visualized with Pop art (http://popart.otago.ac.nz). Automatic barcode gap discovery (ABGD; Puillandre, Lambert, Brouillet, & Achaz, 2012) was used to determine number of molecular operational taxonomic units (mOTU), which were used as basis to assess *Wolbachia* infection status in each mOTU.

### 2.5 *Wolbachia* detection and strain determination

To detect *Wolbachia* and to determine the strain, we used five primers of the Multilocus Sequence Typing System (MLST, https://pubmlst.org/Wolbachia) and the *wsp* locus (Baldo, Hotopp, et al., 2006). In addition, we adopted the ARM (A‐Supergroup repeat motif) primer which were recently developed to detect even weak infection with the *Wolbachia* Supergroup A (Schneider, Klasson, Lind, & Miller, 2014; Tables S2–S4). As a positive control for Supergroup A, we used DNA extracted from *Drosophila suzukii* Matsumura, 1931 (Diptera: Drosophilidae) (Siozios et al., 2013) and for Supergroup B a sample from a Czech *H. postica* population (Iwase, Tani, et al., 2015). PCR conditions for each primer combination followed the original MLST publications (Baldo, Hotopp, et al., 2006). PCR products were purified and sequenced as described above.

**Figure 1:**
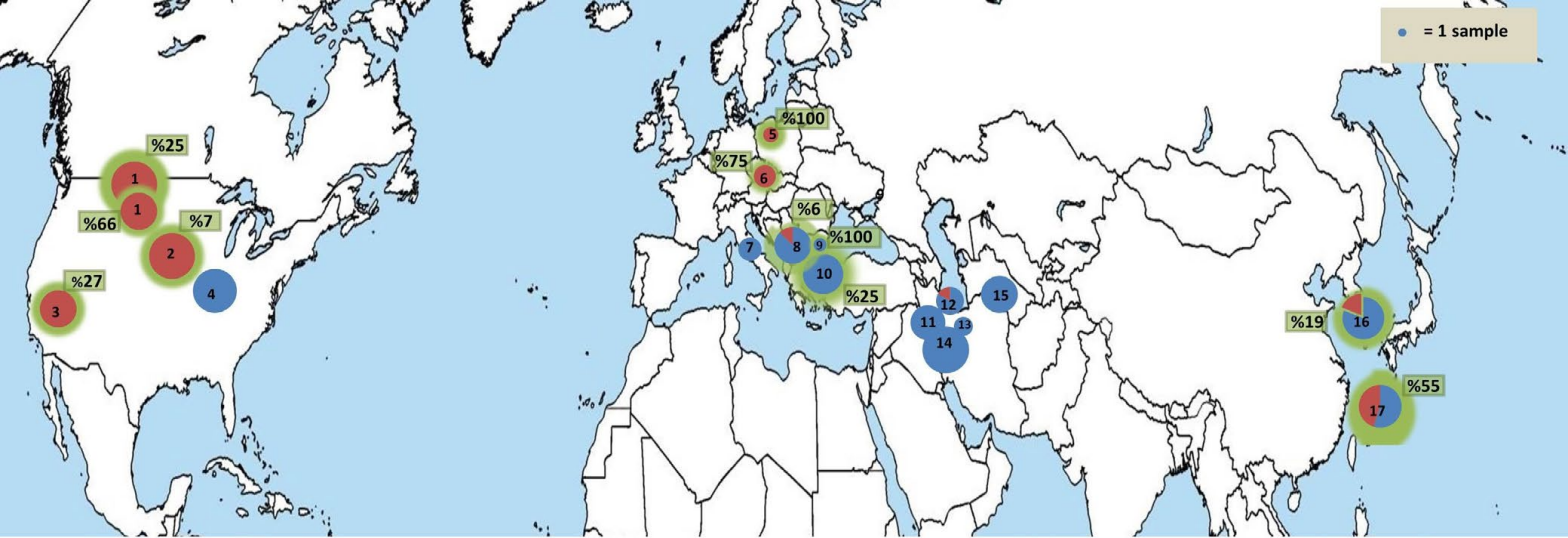
Worldwide distribution of two lineages and *Wolbachia* infection. Each circle represents a population and their area corresponds to the sample size. Blue and red color indicate, respectively, Eastern and Western lineage. Populations with *Wolbachia* infection are indicated with green shadow and infection percentage came into a box. 1. Montana (including two populations in relatively close distance), America, 2. Nebraska, America, 3. California, America, 4. Missouri, America, 5. Poland, 6. Czech, 7. Italy, 8. Knezha, Bulgaria, 9. Lozitsa, Bulgaria, 10. Plovdiv, Bulgaria, 11. Hamedan, Iran, 12. Taleghan, Iran 13. Karaj, Iran, 14. Tuyserkan, Iran, 15. Jovein, Iran, 16. Korea, 17. Japan

**Figure 2:**
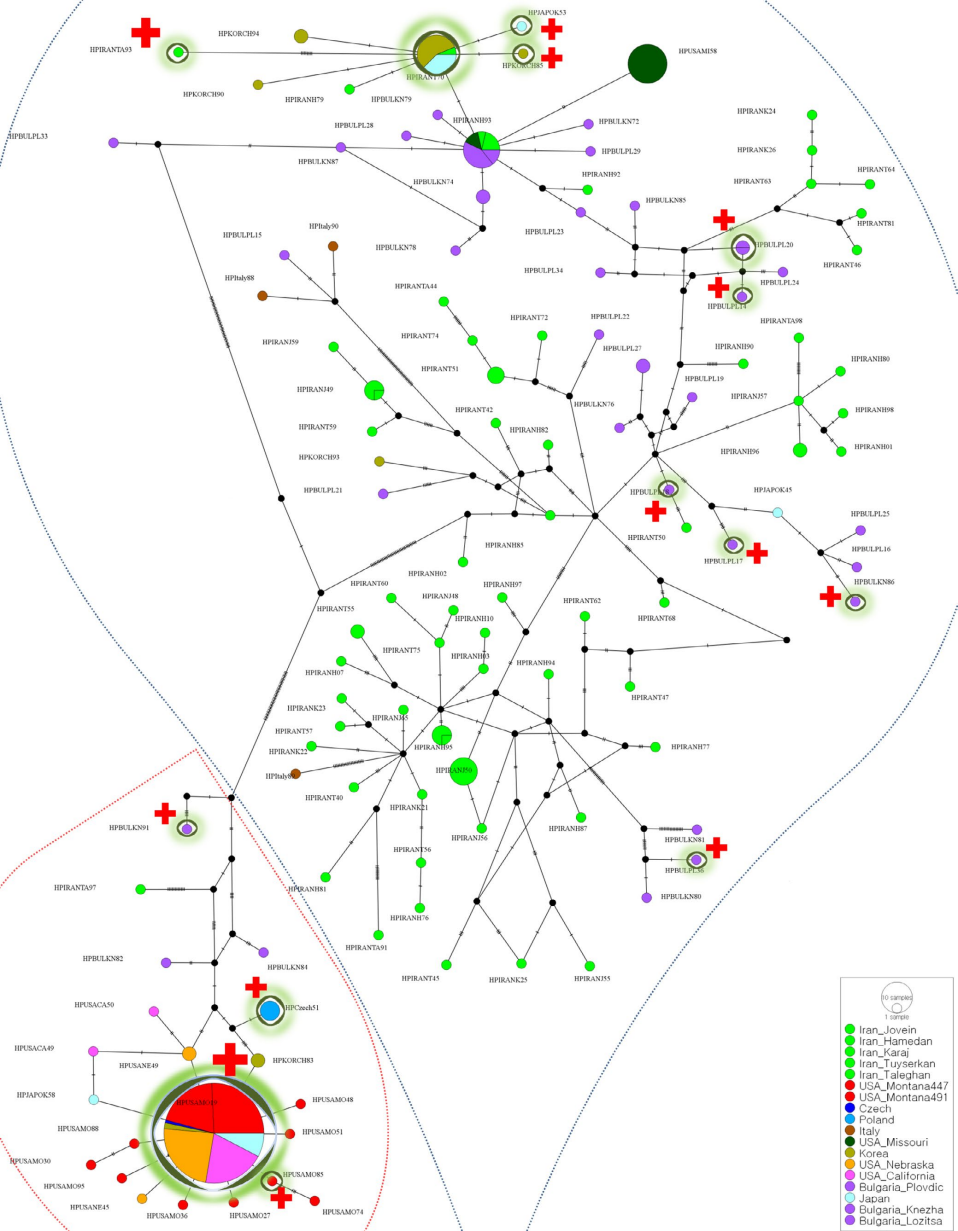
Parsimony haplotype network of COI+CytB sequences with indication of infected haplotypes. Blue and red dotted borders, respectively, grouped Eastern and Western lineage. The haplotypes included at least one infected individual are indicated by a green shade and red + symbol

Based on the MLST protocol (https://pubmlst.org/Wolbachia/), the *Wolbachia* strain type (profile) of each positively infected weevil was determined. We obtained an additional 26 *Wolbachia* sequences for phylogenetic comparison from GenBank and the MLST database in order to confirm the Supergroup of the alfalfa weevil *Wolbachia* strains. (Table S2). Additional sequences were chosen based on the degree of relatedness (most close matches) to our sequences and were further supplemented by several random samples from both Supergroups (Table S2). We then constructed a phylogenetic tree based on a concatenated alignment of all MLST genes (excluding the highly variable wsp gene) using the same methods and settings described above. As *Wolbachia* has a high mutation and recombination rate, we additionally used the ClonalFrameML approach (Didelot & Wilson, 2015), which corrects the branch length and position of taxa to account for possible recombination, in R (v. 3.2.2), to further validate the Supergroup position of the detected strain. The infection frequency for each population was calculated, infected populations were plotted on a map, and infected haplotypes were marked on the haplotype network (Figures 1 and 2).

## 3 RESULTS

### 3.1 Lineage determination and global distribution of the alfalfa weevil

By analyzing 292 alfalfa weevil specimens from Iran, Bulgaria, Italy, Poland, the Czech Republic, western and eastern parts of North America, Korea, and Japan (Appendix S1 and Figure 1), we improved our current knowledge of the biogeography of introduced and native populations of *H. postica*. The analyses of mitochondrial genes (1,776 bp, COI and CytB combined) revealed a deep divergence between the two lineages (average of 90 nucleotide differences, 6.15%; Appendix S3, Table S3, Figure S1). The phylogenetic tree (Figure S2) and the haplotype network (Figure 2) showed that the Eastern lineage is present in five Iranian, two Bulgarian (Plovdiv and Knezha), and one Italian population and in Missouri (North America); the Western lineage was detected in three North American (Montana, Nebraska, and California), one Czech, and one Polish population (Figure 1). At two locations, where both lineages were detected usually, the Eastern lineage was more frequent (Korea: 19% Western and 81% Eastern, Bulgaria Knezha: 8% Western and 92% Eastern lineage, Taleghan: 16% Western [only one sample], 84% Eastern). In Japan, both lineages had the same frequency (Figure 1, Table 2).

**Table 2:** Alfalfa weevil genetic diversity based on A. mitochondrial genes and B. CAD gene

Population	L	N	S	H	Hd ± *SD*	K	Pi ± *SD*	V	P
A.									
Iran, Jovein	E	17	40	8	0.772 ± 0.097	9.808	0.0066 ± 0.0012	22	18
Iran, Hamedan	E	26	65	18	0.978 ± 0.018	15.809	0.0107 ± 0.0005	26	39
Iran, Karaj	E	6	35	6	1.000 ± 0.096	15.933	0.0107 ± 0.0023	14	21
Iran, Tuyserkan	E	26	80	23	0.987 ± 0.016	19.160	0.0129 ± 0.0005	28	52
Iran, Taleghan	E + W	6	119	6	1.000 ± 0.096	46.666	0.0316 ± 0.0102	91	28
	E	5	51	5	1.000 ± 0.126	24.300	0.1640 ± 0.0028	34	17
	W	1	–	1	–	–	–	–	–
USA Montana447	W	32	10	6	0.292 ± 0.105	0.683	0.0004 ± 0.0002	9	1
USA Montana491	W	25	7	5	0.300 ± 0.118	0.633	0.0004 ± 0.0002	6	1
Czech	W	4	–	4	–	–	–	–	–
Poland	W	1	–	1	–	–	–	–	–
Italy	E	3	52	3	1.000 ± 0.272	34.666	0.0234 ± 0.0104	52	0
USA, Missouri	E	18	2	2	0.209 ± 0.116	0.418	0.0002 ± 0.0001	0	2
Korea	E + W	21	104	8	0.804 ± 0.079	31.161	0.0211 ± 0.0064	12	92
	E	17	23	6	0.705 ± 0.106	3.073	0.0020 ± 0.0013	21	2
	W	4	3	2	0.833 ± 0.222	1.833	0.0012 ± 0.0003	1	2
USA, Nebraska	W	28	2	3	0.203 ± 0.098	0.208	0.0001 ± 0.0001	1	1
USA, California	W	23	4	3	0.169 ± 0.102	0.426	0.0002 ± 0.0001	3	1
Bulgaria, Plodvic	E	24	99	19	0.960 ± 0.031	16.742	0.0113 ± 0.0022	65	34
Japan, Fukuoka	E + W	20	100	8	0.768 ± 0.069	47.926	0.0324 ± 0.0024	10	90
	E	11	20	6	0.709 ± 0.137	4.036	0.0027 ± 0.0017	18	2
	W	9	1	2	0.222 ± 0.166	0.222	0.0001 ± 0.0001	1	0
Bulgaria, Knezha	E + W	16	125	14	0.983 ± 0.028	32.066	0.0217 ± 0.0066	25	100
	E	14	58	12	0.978 ± 0.035	14.681	0.0099 ± 0.0027	27	31
	W	2	6	2	1.000 ± 0.500	6.000	0.0040 ± 0.0020	6	0
Bulgaria, Lozitsa	W	1	–	1	–	–	–	–	–
Total	–	303							
Total Eastern L		167	189	100	0.972 ± 0.005	18.033	0.0122 ± 0.0006	62	127
Total Western L		130	56	24	0.360 ± 0.056	1.734	0.0011 ± 0.0003	37	19
Total *H. postica*		297	250	124	0.869 ± 0.018	50.850	0.0344 ± 0.0002	68	182

Population	N	S	H	Hd ± *SD*	K	Pi ± *SD*	V	P
B.								
Iran, Jovein	7	25	4	0.810 ± 0.13	9.808	0.028 ±	8	16
Iran, Hamedan	7	21	4	0.714 ± 0.181	8.857	0.004 ± 0.005	10	11
Iran, Tuyserkan	7	35	5	0.900 ± 0.01	14.38	0.035 ± 0.006	16	18
Montana447	12	24	8	0.848 ± 0.01	8.015	0.019 ± 0.004	10	14
Montana491	9	0	1	0	0	0	0	0
Missouri	13	5	2	0.153 ± 0.126	0.769	0.019 ± 0.001	5	0
Korea	6	34	5	0.933 ± 0.122	14.33	0.035 ± 0.009	2	5
Nebraska	3	5	3	1.00 ± 0.272	3.333	0.008 ± 0.003	5	0
California	8	19	4	0.642 ± 0.184	5.750	0.0143 ± 0.007	14	5
Bul, Plodviv	10	28	10	1.00 ± 0.45	10.73	0.026 ± 0.003	8	19
Japan	11	33	9	0.936 ± 0.05	11.52	0.028 ± 0.003	8	25
Bul, Knezha	6	18	5	0.933 ± 0.122	7.866	0.019 ± 0.004	9	9
Total *H. postica*	99	51	44	0.926 ±	10.53	0.026 ±		

### 3.2 Genetic and haplotype diversity

Regardless of the region, populations belonging to the Eastern lineage comprised higher levels of mitochondrial diversity compared to the Western lineage (Table 2A). The highest haplotype diversity was observed in Karaj, Taleghan (Iran), and Italy (H = 1) (all three populations are Eastern lineages; Table 2A). Based on the haplotype network (Figure 2), two haplotypes of the Eastern lineage (HPIRAN93) were globally common and were found in Iran, Bulgaria, and Missouri. In addition, there was one dominant haplotype detected in Japan, Korea, and Iran. The dominant haplotype of the Western lineage was HPUSAMO19 (Figure 2). This haplotype was observed in all Western lineage populations except that from the Czech Republic.

### 3.3 Genetic diversification based on nuclear genes

Due to either the lack of PCR products or only weak amplification success, we were not able to amplify and sequence nuclear genes for all samples. EF1a was only successfully sequenced for 78 samples (Appendix S1). The pairwise distance among haplotypes indicated 1%–4.2% nucleotide differences in the intron and 0.1%–0.5% differences for the exon; the complete sequence showed 0.1%–1.1% differences. However, based on the phylogenetic tree (Figure S3) and haplotype network, no clear geographic pattern of divergence or any level of lineage classification was observed. For the CAD locus, 111 sequences were analyzed (Appendix S1) with 0.07%–5.9% pairwise nucleotide differences. Similar to EF1a, no geographic/lineage pattern of divergence was observed (Figure S4). Patterns of the distribution of genetic diversity supported the results of mitochondrial genes, but at a lower degree (Table 2B). American populations showed lower diversity compared to the other populations. Similar to mitochondrial genes, Eastern lineage populations showed higher genetic diversity compared to the Western lineage.

### 3.4 Population genetic parameters and neutrality test

In Iran, genetic differentiation was low (.21 > Φ_ST_ > .005) (Table 3); the same was true for the two Eastern lineage populations in Bulgaria. In addition, we found low genetic differentiation between Japanese and Korean Eastern lineage (Φ_ST_ = .041), and higher between the Japanese and Koran Western lineage (Φ_ST_ = .244). Low genetic differentiation was also detected among American Western lineage populations. This, however, may at least partially be an artifact due to insufficient sample size; we did not find evidence of genetic differentiation between Western lineage populations from Europe and American Western lineage populations.

**Table 3:** Comparative *F*_st_ between populations

Populations	1	2	3	4	5	6	7	8	9	10	11	12	13	14	15	16	17	18
1. Iran, Jovein																		
2. Iran_Hamedan	0.170																	
3. Iran_Karaj	0.135	0.045																
4. Iran_Tuyserkan	0.157	0.014	0.012															
5. Iran_Taleghan	0.210	−0.005	0.067	−0.017														
6. USA_Montana447	0.941	0.908	0.912	0.891	0.861													
7. USA_Montana449	0.941	0.908	0.912	0.891	0.862	−0.001												
8. Czech	0.941	0.908	0.911	0.890	0.861	0.913	0.916											
9. Italy	0.451	0.404	0.410	0.382	0.354	0.823	0.823	0.823										
10. USA_Missouri	0.797	0.570	0.621	0.545	0.355	0.993	0.994	0.993	0.630									
11. Korea_Eastern	0.718	0.450	0.512	0.429	0.236	0.979	0.979	0.978	0.579	0.600								
12. Korea_Western	0.935	0.902	0.906	0.884	0.855	0.210	0.214	0.8	0.818	0.987	0.972							
13. USA_Nebraska	0.944	0.911	0.914	0.893	0.864	0.010	0.014	0.941	0.825	0.996	0.981	0.227						
14. USA_California	0.942	0.91	0.913	0.892	0.863	0.010	0.013	0.927	0.824	0.995	0.980	0.206	−0.018					
15. Bulgaria_Plovdiv	0.425	0.148	0.251	0.158	0.012	0.904	0.904	0.904	0.407	0.352	0.242	0.898	0.906	0.905				
16. Japan_Eastern	0.690	0.412	0.480	0.395	0.201	0.973	0.973	0.973	0.562	0.524	−0.041	0.967	0.976	0.975	0.193			
17. Japan_Western	0.944	0.911	0.914	0.893	0.864	0.004	0.007	0.941	0.825	0.996	0.981	0.244	0.012	−0.017	0.906	0.976		
18. Bulgaria.Knezha (E)	0.486	0.211	0.307	0.219	0.045	0.915	0.915	0.914	0.454	0.262	0.160	0.908	0.917	0.916	−0.003	0.114	0.917	
19. Bulgaria.Knezha (W)	0.910	0.877	0.882	0.860	0.831	0.544	0.546	0.487	0.794	0.964	0.949	0.494	0.554	0.543	0.874	0.943	0.562	0.884

AMOVA supported the results of Φ_ST_ estimates and showed that <2% of the genetic variance was found among populations (see among population comparison in Table 4). 76.50% of the variance was attributable to differences between Western and Eastern lineages. Significant differences between Iranian and western American and between Bulgaria and the western United States were observed. Regardless of the geographic distribution, Eastern populations have more genetic similarity to each other rather than a Western population (even in a same location). The western American population was also similar to the western lineage of Eastern Asia (<1% of differences among groups). The divergence of the eastern American lineage and Eastern Asian populations explained 52% of variance.

**Table 4:** AMOVA among geographic groups

	Iran E		USA W		USA E		East Asia E		East Asia W	
	Var%	FI	Va%	FI	Va%	FI	Va%	FI	Va%	FI
USA(W)										
Among group	40.23	**0.402**								
Among population	3.04	**0.051**								
Within population	56.73	**0.432**								
USA(E)										
Among group	27.99	0.279	76.60	0.765						
Among population	5.61	**0.077**	−0.23	**−0.009**						
Within population	66.39	**0.336**	23.72	0.762						
East Asia (E)										
Among group	12.74	0.127	61.88	0.618	52.73	0.527				
Among population	4.12	**0.472**	−0.59	−0.015	−1.29	−0.027				
Within population	83.13	**0.168**	38.71	**0.612**	48.56	**0.514**				
East Asia (W)										
Among group	21.65	0.216	0.79	0.007	62.16	0.621	41.95	0.419		
Among population	4.83	**0.061**	1.31	0.013	9.50	0.251	−0.49	−0.008		
Within population	73.52	**0.264**	97.90	0.020	28.34	**0.716**	58.54	0.414		
Bulgaria (E)										
Among group	−0.15	−0.001	48.46	0.484	33.55	0.335	17.31	0.173	25.77	0.257
Among population	4.50	**0.044**	−0.30	−0.005	1.10	0.016	−1.41	−0.170	0.29	0.003
Within population	95.65	**0.043**	51.58	**0.481**	65.35	**0.346**	84.10	**0.159**	73.94	**0.260**

Note

W = Western and E = Eastern linage; Iran E (Jovein, Hamedan, Karaj, Tuyserkan, Taleghan), USA W (Montana, Nebraska, California), USA E (Missouri), East Asia E (Eastern lineage of Korea and Japan), East Asia W (Western lineage of Korea and Japan) and Bulgaria E (Plovdiv, Knezha). Percentage of diversity distributed among geographic groups, among populations in each geographic group and the individual one within each population as well as fixation index (FI) is given. The significant *p*‐value (*p* < 0.05) is bolded.

Tajima's D, Fu's F, and Li's D tests on the mitochondrial genes (Table 5A) rejected neutrality for both Montana populations suggesting a selective sweep or bottleneck may have taken place. However, based on the nuclear genes, only the Missouri population is experiencing a bottleneck (Table 5B). According to HKA and McDonald–Kreitman tests on mitochondrial genes, no selective pressure was detected on the Western lineage, nor on *Wolbachia* infected populations of the Eastern lineage (Bulgaria) (Table S5). Similarly, dN/dS ratio tests found no significant adaptation or selection on mitochondrial genes in the Western and Eastern lineages (ω = .0423, *p* value: .439, and ω = .0622, *p* value: .236, respectively).

**Table 5:** Tajima D (TD) and Fu and Li's D (FLD) on A. mitochondrial genes and B. CAD gene

Population	L	TD	Sig (*p*)	FLD	*p* value
A					
Iran‐Jovein	E	−0.70701	>.10	−1.24954	>.10
Iran‐Hamedan	E	−0.27770	>.10	−0.91294	>.10
Iran‐Karaj	E	0.25098	>.10	0.41263	>.10
Iran‐Tuyserkan	E	−0.33477	>.10	−0.65375	>.10
Iran‐Taleghan	E + W	−0.67684	>.10	−0.82602	>.10
	E	−0.33843	>.10	−0.24937	>.10
	W	–	–	–	–
USA Montana‐1	W	−2.26318	<.01*	−3.52861	<.02*
USA Montana‐2	W	−2.03576	<.05*	−2.70693	<.05*
USA Missouri	E	−0.68482	>.10	0.88460	>.10
Korea	E + W	0.27588	>.10	1.03369	>.10
	E	−2.19381	<.01*	−3.03208	<.02*
	W	1.08976	>.10	1.08976	>.10
USA Nebraska	W	−1.24137	>.10	−0.71444	>.10
USA California	W	−1.67904	.10 > *p *> .05	−1.79568	>.10
Bulgaria Plodvic	E	−1.48703	>.10	−2.49790	>.10
Japan	E + W	2.87706	<.01*	1.17882	<.02*
	E	−1.85247	<.05*	−2.16730	<.05*
	W	−1.08823	>.10	−1.18990	>.10
Bulgaria Knezha	E + W	−0.73088	>.10	0.59500	>.10
	E	−0.91989	>.10	−0.66893	>.10
	W	–	–	–	–
Total Eastern	E	−2.77975	<.05*	−2.77975	<.05*
Total Western	W	−2.61643	<.02*	−6.07163	<.01*
B					
Iran‐Jovein	E	0.41349	>.10	0.58458	>.10
Iran‐Hamedan	E	0.18757	>.10	0.04642	>.10
Iran‐Tuyserkan	E	−0.12217	>.10	0.01385	>.10
USA Montana−1	W	0.03792	.03792	−0.24142	>.10
USA Missouri	E	−1.86311	<.05*	−2.32348	<.05*
Korea	E + W	−0.23782	>.10	−0.30248	>.10
USA Nebraska	W	−1.78190	.10 > *p *> .05	−1.81894	>.10
Bulgaria Plodvic	E	0.22555	>.10	0.08206	>.10
Japan	E + W	0.28633	>.10	0.51507	>.10
Bulgaria Knezha	E + W	−0.01305	>.10	0.08206	>.10
Total Eastern	E	−0.65952	>.10	−1.34371	>.10
Total Western	W	−0.37444	>.10	−0.64136	>.10

Note

Significance is indicated by

* *p* < .05.

### 3.5 Phylogenetic analysis

**Figure 3:**
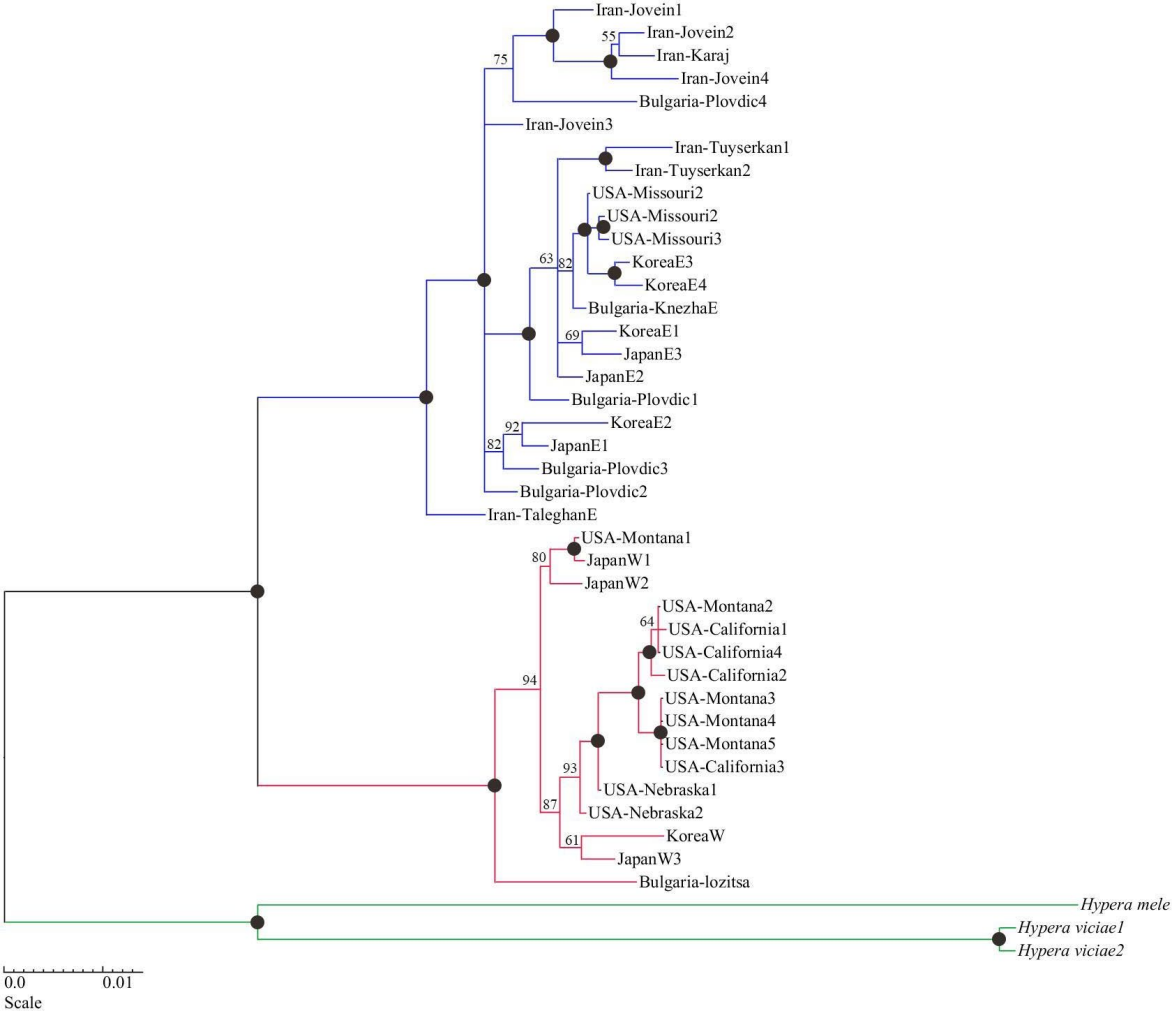
Concatenated Bayesian tree based on mitochondrial genes (CytB and COI) and nuclear genes (Ef1a and CAD). Each node number represents the posterior probability value. The blue line is indicator for Eastern lineage, red line for Western lineage, green line for out‐groups, and black circle is the posterior probability value above 95%

Nuclear genes were compatible with mitochondrial genes (partition‐homogeneity test *p*‐value = .17) and were used jointly to construct a phylogenetic tree. In addition to the high support of each lineage, Western lineage sample of Lozitsa (Bulgaria) formed a single supportive clade against other Western lineage samples (Figure 3). This can be an indication of intralineage differentiation.

**Figure 4:**
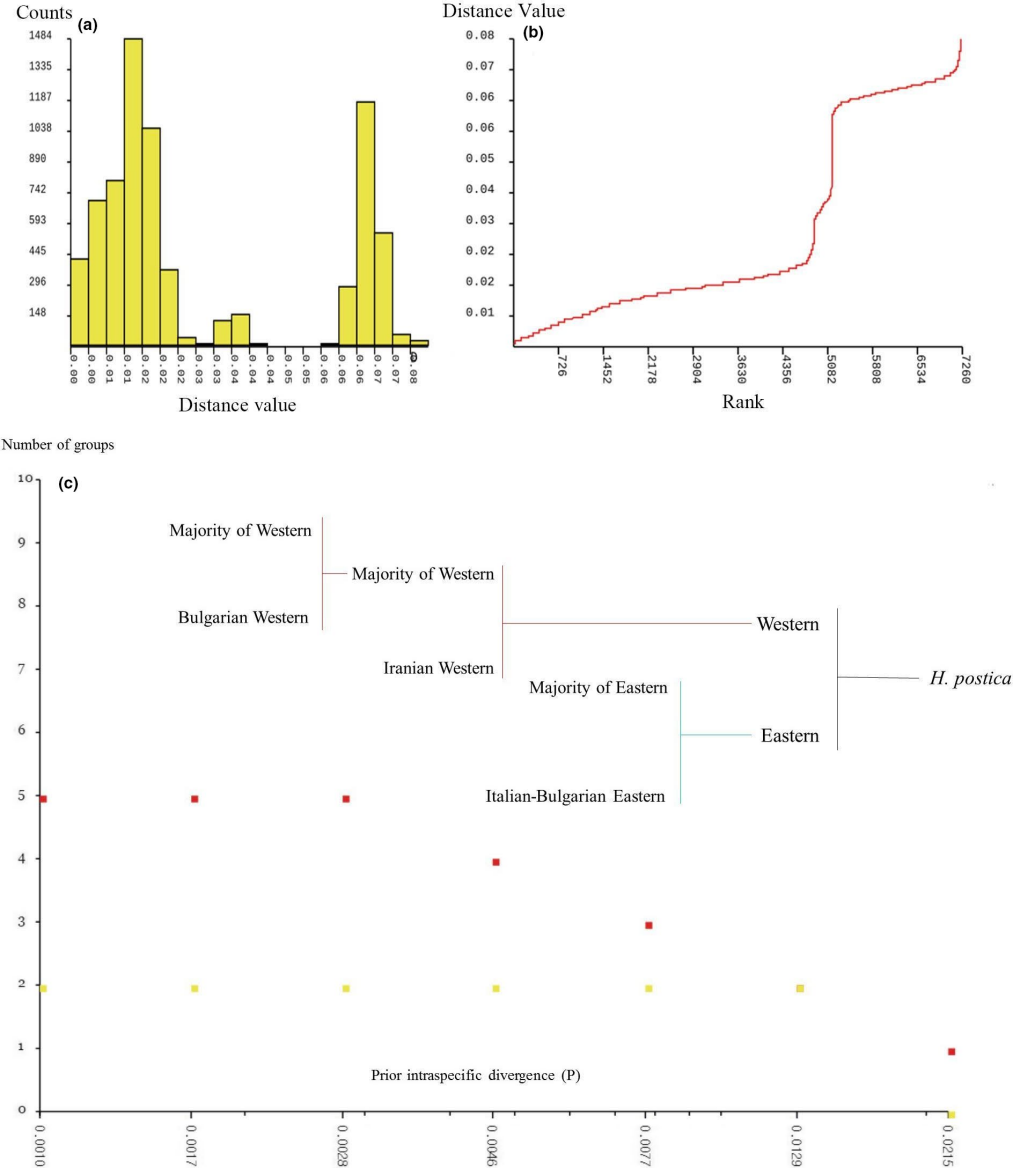
ABGD for mitochondrial genes. Maximum 5 molecular taxonomic units inferred with ABGD are presented. (a) The frequency of divergence classes across all samples (without out‐groups) a clear divergence is observed. (b) The distance value is ranked and showed the gap from 2% to 6% distance. (c) The numbers of groups detected by ABGD depending on the prior intraspecific threshold, the number of groups started from two major groups (Western‐Eastern) at 0.0129 P, so more than this value we expect only two groups until 0.215 P. The third group (Italian Bulgarian) appeared at *p* = .007 threshold, fourth group (Iranian Western) at *p* = .0046, and finally the last group is detected at *p* = .0028 and less than that. Red spot: recursive partition, yellow spot: initial partition

The ABGD analysis detected a maximum four mOTUs. The best supported groups with sufficient prior interspecific divergence (P) (*p* > .0129) were the Western and Eastern lineages with a deep gap (Figure 4a,b). However, when decreasing the P threshold, more groups were detected (Figure 4c). The Bulgarian Italian samples represented the first splitting from the Eastern lineage with an average of 3.1% nucleotide differences from the other Eastern lineage members (HPItaly88, HPItaly90, and HPBulPL15). In the Western lineage, two distinct singleton mOTUs were observed: (a) a single Iranian sample (HPIRANTA97) and (b) a sample from Bulgaria‐Lozitsa (HPBULLO91; Figure 2).

### 3.6 *Wolbachia* detection and strain determination

Except for samples of populations from Iran, Italy and Missouri and *H. meles*, *Wolbachia* infection was detected in all other populations and out‐group species with variable infection rates (Table 1, Figure 1). The infection rate varied from 66% in a Montana population to 6% in Knezha (Bulgaria) (Figure 1). HPUSAMO19 was also a commonly infected haplotype from Japanese to American populations (Figure 2). In the Eastern lineage, the most commonly infected haplotype was found in Japan and Korea. However, in Bulgaria, every single infection appeared with a unique host haplotype.

PCR products of ARM primers showed strong infection in positive controls, but not in our samples, indicating a *Wolbachia* strain different from Supergroup A. The ftsZ and hcpA genes could not be amplified for most of the samples. From a total number of 59 samples, we successfully amplified gatB (corresponding to the total number of infected samples) and among them, coxA, fbpA, and wsp were successfully amplified for 35 specimens. Analyses of the sequence data suggested that three different *Wolbachia* strains of Supergroup B infected the samples. The first one (subsequently named *W*Hypera1) was the predominant one and appeared in 57 samples. *W*Hypera2 was detected in one sample from California differs only by one base pair from *W*Hypera1 (gatB gene). *W*Hypera3 was detected in a sample of the America‐Montana population (HP6033) and has 5.6% and 6.9% difference for coxA, and fbpA and only one base per difference for gatB.

The wsp gene was successfully amplified for all samples and represents the most variable locus. The chromatograms of sequenced samples further suggested coinfection with both *w*Hypera1 and *w*Hypera3 in one sample. However, based on the limitation of our methodology, we can only suggest the possibility of double infection. The most frequent *Wolbachia* types *w*Hypera1 and *w*Hypera2 have not been previously reported (based on blast in both GenBank and MLST database in 01.05.2019). The single gatB sequence of *w*Hypera1 we were able to generate is compatible with MLST allele number 9. The Bayesian tree based on three MLST genes, supported by ClonalFrameML approach (Figure S5), confirmed the phylogenetic position of these three *Wolbachia* types of *H. postica* in Supergroup B.

## 4 DISCUSSION

### 4.1 A more comprehensive picture of genetic diversity and lineage distribution of alfalfa weevil

By generating comprehensive molecular data covering invasive and native populations of the alfalfa weevil and the type of *Wolbachia* infection, we increased the current knowledge of this cosmopolitan pest species and its *Wolbachia* infection. We detected a deep mitochondrial split between the two main lineages that are now globally distributed. The Eastern lineage demonstrates a higher level of diversity and is dominant in the Mediterranean basin, the Middle East, Eastern Europe, and the eastern America. The less diverse Western lineage was detected mainly in central and western Europe and the western America. Moreover, both lineages coexisted in Eastern Asia, and a weak coexistence with Eastern lineage dominance was observed in the Middle East and Eastern Europe.

Within the Eastern lineage, all Iranian and Bulgarian populations are diversified both in nuclear and mitochondrial genes (Table 2). In this region, low genetic differentiation, which can be a sign of gene flow (whether by human‐mediated dispersal [Sanaei et al., 2016] or natural migration [Prokopy & Gyrisco, 1965]), may explain their rich gene pool. However, the high genetic diversity typical for of the Eastern lineage was not observed in some of the introduced populations (e.g., Missouri; Table 1). Such reduced genetic diversity is typical for leading edge and introduced populations due to founder effects.

The Western lineage has a much lower genetic diversity in all populations including introduced and native distributions (Table 2). Similar to the Eastern lineage, strong gene flow was suggested for the Western lineage, particularly within American populations. While the Eastern lineage is predominant in Iran and Bulgaria, the Western lineage occurs in these regions at a low frequency. Iranian and Bulgarian Western lineage specimens were the most distinct samples within the Western lineage (Figures 3 and 4). There is no trace of these distinct genetic variants in Central Europe, North America, and Eastern Asia (Figure 2, Table 3). This suggests some local differentiation, but also shows that these were not the source of introduction in the non‐native regions.

### 4.2 Type and degree of *Wolbachia* infection in the alfalfa weevil

Past studies suggested that resistance to *Wolbachia* evolved in the Eastern lineage of *H. postica* (Hsaio, 1996). However, according to the best of our knowledge, there is less evidence to support the existence of resistance to all *Wolbachia* strains in a particular arthropod species (Weinert, Araujo‐Jnr, Ahmed, & Welch, 2015). In support of recent findings (Iwase, Tani, et al., 2015), our results refute the classical view indicating that the Eastern lineage generally is resistant to infection. We found infection in Eastern lineage samples from Bulgaria, Korea, and Japan; hence, our data show that the infection is not or only little correlated to the lineage type, as all populations of *H. postica* are probably vulnerable to infection. However, infection appears to not have occurred in the Eastern lineage populations in the Middle East, eastern North America, and Italy or the parasite has recently been lost without clear pattern of DNA variation in mitochondrial DNA. Yet, one problem may be the specificity of MLST, which does not allow to detected new strains and our sequence data suggested some divergence from the commonly detected Supergroup strains.

*Wolbachia* host shift, which is also referred as horizontal transmission, is not a rare event in beetles and other arthropods (Bailly‐Bechet et al., 2017; Chrostek, Pelz‐Stelinski, Hurst, & Hughes, 2017; Lachowska, Kajtoch, & Knutelski, 2010; Tolley, Nonacs, & Sapountzis, 2019; Yun, Peng, Liu, & Lei, 2011). Generally, it is common to find various types of strains (*Wolbachia* diversity) resulting from recent host shifts in a cosmopolitan host species, rather than a uniform infection of a certain strain across the host's range (Ali et al., 2018; Avtzis, Doudoumis, & Bourtzis, 2014; Chen et al., 2017; Chen, Zhang, Du, Jin, & Hong, 2016; Goryacheva, Blekhman, Andrianov, Gorelova, & Zakharov, 2015; Huchesh & Puttaraju, 2014; Jiang, Wu, He, Zhu, & Yu, 2016; Mariño, Verle Rodrigues, & Bayman, 2017). Our data suggest that in the alfalfa weevil, *w*Hypera1 is the predominant strain of *Wolbachia* across all populations. This strain has not been reported from other arthropod hosts so far. A variant of the predominant *Wolbachia* strain is *w*Hypera2, which is the only other variant of *w*Hypera1 that we found in a large number of samples. *wHypera3* is another rare *Wolbachia* infection, which is observed as a coinfection with *w*Hypera1 in a sample from the Montana population. This rare strain may be a coincidental infection and has not become established in the population. Such a rare and unexpected *Wolbachia* infection type was previously reported from Japan (Iwase & Tani, 2016; Iwase, Tani, et al., 2015). We also found the *w*Hypera1 strain in *H. viciae*, a closely related species of the alfalfa weevil. The “phylogenetic distance effect” is a hypothesis predicting a decline in transmission chance of pathogens/symbionts with increasing genetic distance between donor and recipient (Charleston & Robertson, 2002; Engelstädter & Hurst, 2006). Even though in most case studies, there is no correlation between *Wolbachia* phylogeny and weevil host systematics at the level of subfamily and tribes (Kajtoch & Kotásková, 2018), there are few examples supporting a transmission of closely related *Wolbachia* strains between different species (Lachowska et al., 2010). The two closely related species (*H. postica* and* H. vicieae*) were infected with the same *Wolbachia* strain. *W*Hypera1 might be found more widely within *Hypera* and other related genera. However, it is also possible that *Wolbachia* is horizontally transmitted to *H. vicieae* as they both can coexist in the same fields.

### 4.3 Effect of *Wolbachia* on the alfalfa weevil genetic diversity

The low genetic diversity of the Western lineage can be an intrinsic character of this lineage. The recent evolved clades usually are observed with lower genetic diversity compare to the elder (ancestor) clade (Tilmon, 2008). Although our data (especially nuclear genes) and analyses cannot confirm or reject the position of the Eastern lineage as the ancestral, the reason of the low genetic diversity of the Western lineage may be found in the young age of this lineage (or other historical elements) rather than a recent genetic reduction. In the other plausible scenario, genetic diversity reduction might be caused by *Wolbachia* drives or demographic events.

*Wolbachia* and the beetle hosts may have an unstable relationship leading to the lack of a clear effect of *Wolbachia* infection on the host's ecology and phylogeny (Kajtoch et al., 2019). However, even in a short period of infection, mitochondrial hitchhiking effects driven by *Wolbachia* are commonly observed in beetles (Ali et al., 2018; Arthofer, Avtzis, Riegler, & Stauffer, 2010; Jäckel, Mora, & Dobler, 2013; Mazur et al., 2016) and other arthropods (Avtzis et al., 2014; Cariou, Duret, & Charlat, 2017; Narita et al., 2006; Rousset, Vautrin, & Solignac, 1992; Shoemaker, Dyer, Ahrens, McAbee, & Jaenike, 2004).

However, in many case studies, demographic events, such as bottlenecks, rather than *Wolbachia* infection seem to be a likely explanation for reduced genetic diversity; yet, these effects may be altered in the face of *Wolbachia* infection (Rodriguero, Lanteri, & Confalonieri, 2010; Rokas, Atkinson, Brown, West, & Stone, 2001). However, there are also some empirical examples in arthropods, where *Wolbachia* actually drives the decrease of mitochondrial genetic diversity via sweeping effects (Chen et al., 2016).

The question remains whether *Wolbachia* has caused the reduced mitochondrial diversity in the Western lineage. We cannot clearly answer this question with our data, but suggest that demographic effects rather than *Wobachia* have driven this reduced diversity. This is supported by several observations: Firstly, we observed that populations with low genetic diversity (e.g., Nebraska), but also, such with high genetic diversity (e.g., Bulgaria) may be infected with *Wolbachia*. Further, we did not detect any signs of selection on mitochondrial DNA in most populations regardless of their genetic diversity and lineage (Table 5). Finally, no signs of selection was detected in neither Western nor Eastern lineages. Therefore, there appears to be no clear correlation between *Wolbachia* infection and the genetic diversity of the alfalfa weevil at most of the locations we studied. Hence, we suggest that the low genetic diversity noted for some *H. postica* populations is more likely the result of historical demographic events (i.e., founder effects and bottlenecks) rather than that of *Wolbachia* infection.

However, in a few populations, our data suggest that *Wolbachia* may have had a negative effect on genetic diversity. Among populations with sufficient individuals, the two Montana populations have one of the highest infection rates (Table 1, Figure 1). Neutrality test based on mitochondrial genes was rejected for these populations without any trace in the nuclear genes (Table 5). Therefore, the high infection rate of *Wolbachia* may have caused linkage disequilibrium between mitochondrial and nuclear genes. Such *Wolbachia* effects may well explain the low genetic diversity in the Montana population. While the two mentioned populations may have suffered genetic reduction by *Wolbachia*, other populations with *Wolbachia* infection did not show reduced genetic diversity and infection rates were similar independent of the host haplotype (Figure 2).

## 5 CONCLUSION

The comprehensive molecular data, which is generated in this study, provided a better picture of the global distribution of both alfalfa weevil and its *Wolbachia* endosymbiont diversity. In addition, we found that regardless of lineage and population, most of the infection of alfalfa weevils are caused by a common strain of *Wolbachia* named *w*Hypera1. However, high mitochondrial genetic diversity of the Eastern lineage and the low diversity in the Western lineage may be explained better by demographic events rather than *Wolbachia* infection. The data generated in this study may provide a useful basis for future studies on alfalfa weevil evolution, but may also provide some information for pest management.

## CONFLICT OF INTEREST

The authors declare that they have no competing interests.

## AUTHOR CONTRIBUTIONS

ES, IK, and MH conceived the idea. ES and MJK performed molecular analyses including DNA extractions, PCRs, and the preparation of the raw data. ES, MS, MR, MT, TBT, and DA performed fieldwork and collecting materials. ES, IK, MH, and MJK performed phylogenetic, population genetic, and neutrality test analysis. ES, MH, MS, MR, MT, and TBT contributed to writing of the manuscript, and the final version was prepared by ES. All authors read and approved the final version of the manuscript.

## Data Availability

The datasets supporting the conclusions of this article are included within the article and its additional supporting file (Appendix S1) where you can find the accession numbers of the GenBank repository.
