# Supplementary material for: Global genetic diversity, lineage distribution, and Wolbachia infection of the alfalfa weevil Hypera postica (Coleoptera: Curculionidae)
Source: Ecol Evol. 2019 Aug 6;9(17):9546–63. doi: 10.1002/ece3.5474 (PMC6745856; doi:10.1002/ece3.5474)
Supplement: Supplementary file 1 [file ECE3-9-9546-s001.pdf]

A comprehensive dataset generated in this study. The Genbank accession number for each sample/gene as well as haplotype group and *Wolbachia* status are provided

Sample code	Species	Population	COI	CytB	Mt Lineage	MtDNA haplotype	Elfa	Ef1a haplotype	CAD	CAD haplotype	*Wolbachia* Infection	coxA	fbpA	gatB	wasp	Strain
BZ6590	*Brachypera zolilus*	Lozitsa, Bulgaria	KY796641	Yes		*B. zoilus*			KY796343	*B. zoilus*	Yes			KY797089		*w* Hypera1
HM5943	*Hypera mele*	Taleghan, Iran	KY796424	KY796721	KY796938	*H. mele*	KY796962	*H. mele*	KY796257	*H. mele*	No			KY797147		
HP5748	*Hypera postica*	Jovein, Iran	KY796349	KY796646	Eastern	HPIRANJ48			KY796237	HPIRANJ48	No					
HP5749	*Hypera postica*	Jovein, Iran	KY796350	KY796647	Eastern	HPIRANJ49					No					
HP5750	*Hypera postica*	Jovein, Iran	KY796351	KY796648	Eastern	HPIRANJ50					No					
HP5751	*Hypera postica*	Jovein, Iran	KY796352	KY796649	Eastern	HPIRANJ50	KY796943	HPIRANJ51			No					
HP5752	*Hypera postica*	Jovein, Iran	KY796353	KY796650	Eastern	HPIRANJ50	KY796944	HPIRANJ51	KY796238	HPIRANJ52	No					
HP5754	*Hypera postica*	Jovein, Iran	KY796354	KY796651	Eastern	HPIRANJ50	KY796945	HPIRANJ54			No					
HP5755	*Hypera postica*	Jovein, Iran	KY796355	KY796652	Eastern	HPIRANJ55	KY796946	HPIRANJ55			No					
HP5756	*Hypera postica*	Jovein, Iran	KY796356	KY796653	Eastern	HPIRANJ56	KY796947	HPIRANJ56	KY796239	HPIRANJ56	No					
HP5757	*Hypera postica*	Jovein, Iran	KY796357	KY796654	Eastern	HPIRANJ57	KY796948	HPIRANJ54	KY796240	HPIRANJ57	No					
HP5758	*Hypera postica*	Jovein, Iran	KY796358	KY796655	Eastern	HPIRANJ50					No					
HP5759	*Hypera postica*	Jovein, Iran	KY796359	KY796656	Eastern	HPIRANJ59					No					
HP5760	*Hypera postica*	Jovein, Iran	KY796360	KY796657	Eastern	HPIRANJ49	KY796949	HPIRANJ60	KY796241	HPIRANJ56	No					
HP5761	*Hypera postica*	Jovein, Iran	KY796361	KY796658	Eastern	HPIRANJ50					No					
HP5762	*Hypera postica*	Jovein, Iran	KY796362	KY796659	Eastern	HPIRANJ50			KY796242	HPIRANJ52	No					
HP5763	*Hypera postica*	Jovein, Iran	KY796363	KY796660	Eastern	HPIRANJ50					No					
HP5764	*Hypera postica*	Jovein, Iran	KY796364	KY796661	Eastern	HPIRANJ49					No					
HP5765	*Hypera postica*	Jovein, Iran	KY796365	KY796662	Eastern	HPIRANJ65					No					
HP5776	*Hypera postica*	Hamedan, Iran	KY796366	KY796663	Eastern	HPIRANH76			KY796244	HPIRANJ56	No					
HP5777	*Hypera postica*	Hamedan, Iran	KY796367	KY796664	Eastern	HPIRANH77			KY796243	HPIRANJ56	No					
HP5779	*Hypera postica*	Hamedan, Iran	KY796368	KY796665	Eastern	HPIRANH79			KY796245	HPIRANJ52	No					
HP5780	*Hypera postica*	Hamedan, Iran	KY796369	KY796666	Eastern	HPIRANH80			KY796246	HPIRANH80	No					
HP5781	*Hypera postica*	Hamedan, Iran	KY796370	KY796667	Eastern	HPIRANH81					No					
HP5782	*Hypera postica*	Hamedan, Iran	KY796371	KY796668	Eastern	HPIRANH82					No					
HP5785	*Hypera postica*	Hamedan, Iran	KY796372	KY796669	Eastern	HPIRANH85					No					
HP5787	*Hypera postica*	Hamedan, Iran	KY796373	KY796670	Eastern	HPIRANH87					No					
HP5790	*Hypera postica*	Hamedan, Iran	KY796374	KY796671	Eastern	HPIRANH90	KY796950	HPIRANH90			No					
HP5792	*Hypera postica*	Hamedan, Iran	KY796375	KY796672	Eastern	HPIRANH92					No					
HP5793	*Hypera postica*	Hamedan, Iran	KY796376	KY796673	Eastern	HPIRANH93					No					
HP5794	*Hypera postica*	Hamedan, Iran	KY796377	KY796674	Eastern	HPIRANH94					No					
HP5795	*Hypera postica*	Hamedan, Iran	KY796378	KY796675	Eastern	HPIRANH95	KY796951	HPIRANH95			No					
HP5796	*Hypera postica*	Hamedan, Iran	KY796379	KY796676	Eastern	HPIRANH96	KY796952	HPIRANJ51			No					
HP5797	*Hypera postica*	Hamedan, Iran	KY796380	KY796677	Eastern	HPIRANH97					No					
HP5798	*Hypera postica*	Hamedan, Iran	KY796381	KY796678	Eastern	HPIRANH98	KY796953	HPIRANH98	KY796247	HPIRANJ56	No					
HP5799	*Hypera postica*	Hamedan, Iran	KY796382	KY796679	Eastern	HPIRANH95					No					
HP5801	*Hypera postica*	Hamedan, Iran	KY796383	KY796680	Eastern	HPIRANH01			KY796248	HPIRANJ56	No					
HP5802	*Hypera postica*	Hamedan, Iran	KY796384	KY796681	Eastern	HPIRANH02					No					
HP5803	*Hypera postica*	Hamedan, Iran	KY796385	KY796682	Eastern	HPIRANH03					No					
HP5804	*Hypera postica*	Hamedan, Iran	KY796386	KY796683	Eastern	HPIRANH96					No					
HP5805	*Hypera postica*	Hamedan, Iran	KY796387	KY796684	Eastern	HPIRANH95					No					
HP5806	*Hypera postica*	Hamedan, Iran	KY796388	KY796685	Eastern	HPIRANH93			KY796249	HPIRANH06	No					
HP5807	*Hypera postica*	Hamedan, Iran	KY796389	KY796686	Eastern	HPIRANH07					No					
HP5809	*Hypera postica*	Hamedan, Iran	KY796390	KY796687	Eastern	HPIRANH93					No					
HP5810	*Hypera postica*	Hamedan, Iran	KY796391	KY796688	Eastern	HPIRANH10					No					
HP5821	*Hypera postica*	Karaj, Iran	KY796392	KY796689	Eastern	HPIRANK21	KY796954	HPIRANJ54			No					
HP5822	*Hypera postica*	Karaj, Iran	KY796393	KY796690	Eastern	HPIRANK22					No					
HP5823	*Hypera postica*	Karaj, Iran	KY796394	KY796691	Eastern	HPIRANK23					No					
HP5824	*Hypera postica*	Karaj, Iran	KY796395	KY796692	Eastern	HPIRANK24					No					
HP5825	*Hypera postica*	Karaj, Iran	KY796396	KY796693	Eastern	HPIRANK25	KY796955	HPIRANK25	KY796250	HPIRANJ56	No					
HP5826	*Hypera postica*	Karaj, Iran	KY796397	KY796694	Eastern	HPIRANK26			KY796251	HPIRANJ57	No					
HP5839	*Hypera postica*	Tuyserkan, Iran	KY796398	KY796695	Eastern	HPIRANJ49					No					
HP5840	*Hypera postica*	Tuyserkan, Iran	KY796399	KY796696	Eastern	HPIRANT40					No					
HP5842	*Hypera postica*	Tuyserkan, Iran	KY796400	KY796697	Eastern	HPIRANT42					No					

HP5845	*Hypera postica*	Tuyserkan, Iran	KY796401	KY796698	Eastern	HPIRANT45					No					
HP5846	*Hypera postica*	Tuyserkan, Iran	KY796402	KY796699	Eastern	HPIRANT46					No					
HP5847	*Hypera postica*	Tuyserkan, Iran	KY796403	KY796700	Eastern	HPIRANT47					No					
HP5850	*Hypera postica*	Tuyserkan, Iran	KY796404	KY796701	Eastern	HPIRANT50					No					
HP5851	*Hypera postica*	Tuyserkan, Iran	KY796405	KY796702	Eastern	HPIRANT51	KY796956	HPIRANT51			No					
HP5853	*Hypera postica*	Tuyserkan, Iran	KY796406	KY796703	Eastern	HPIRANT51					No					
HP5855	*Hypera postica*	Tuyserkan, Iran	KY796407	KY796704	Eastern	HPIRANT55			KY796252	HPIRANT55	No					
HP5856	*Hypera postica*	Tuyserkan, Iran	KY796408	KY796705	Eastern	HPIRANT56					No					
HP5857	*Hypera postica*	Tuyserkan, Iran	KY796409	KY796706	Eastern	HPIRANT57					No					
HP5859	*Hypera postica*	Tuyserkan, Iran	KY796410	KY796707	Eastern	HPIRANT59					No					
HP5860	*Hypera postica*	Tuyserkan, Iran	KY796411	KY796708	Eastern	HPIRANT60					No					
HP5862	*Hypera postica*	Tuyserkan, Iran	KY796412	KY796709	Eastern	HPIRANT62					No					
HP5863	*Hypera postica*	Tuyserkan, Iran	KY796413	KY796710	Eastern	HPIRANT63	KY796957	HPIRANJ55	KY796253	HPIRANT63	No					
HP5864	*Hypera postica*	Tuyserkan, Iran	KY796414	KY796711	Eastern	HPIRANT64	KY796958	HPIRANH98	KY796254	HPIRANJ57	No					
HP5867	*Hypera postica*	Tuyserkan, Iran	KY796415	KY796712	Eastern	HPIRANH95					No					
HP5868	*Hypera postica*	Tuyserkan, Iran	KY796416	KY796713	Eastern	HPIRANT68			KY796255	HPIRANT	No					
HP5870	*Hypera postica*	Tuyserkan, Iran	KY796417	KY796714	Eastern	HPIRANT70					No					
HP5872	*Hypera postica*	Tuyserkan, Iran	KY796418	KY796715	Eastern	HPIRANT72					No					
HP5873	*Hypera postica*	Tuyserkan, Iran	KY796419	KY796716	Eastern	HPIRANT51					No					
HP5874	*Hypera postica*	Tuyserkan, Iran	KY796420	KY796717	Eastern	HPIRANT74					No					
HP5875	*Hypera postica*	Tuyserkan, Iran	KY796421	KY796718	Eastern	HPIRANT75					No					
HP5879	*Hypera postica*	Tuyserkan, Iran	KY796422	KY796719	Eastern	HPIRANT55			KY796256	HPIRANT55	No					
HP5881	*Hypera postica*	Tuyserkan, Iran	KY796423	KY796720	Eastern	HPIRANT81					No					
HP5944	*Hypera postica*	Taleghan, Iran	KY796425	KY796722	Eastern	HPIRANTA44	KY796960	HPIRANJ54	KY796258	HPIRANTA44	No					
HP5946	*Hypera postica*	Warsaw, Poland	KY796483	KY796780	Western	HPUSAMO19					Yes	KY797021	KY797055	KY797090	MK190871	*w* Hypera1
HP5948	*Hypera postica*	Chraberce, Czechia	KY796484	KY796781	Western	HPCzech51					No					
HP5951	*Hypera postica*	Chraberce, Czechia	KY796485	KY796782	Western	HPCzech51					Yes	KY797022	KY797056	KY797091	MK190872	*w* Hypera1
HP5952	*Hypera postica*	Chraberce, Czechia	KY796486	KY796783	Western	HPCzech51					Yes	KY797023	KY797057	KY797092	MK190873	*w* Hypera1
HP5953	*Hypera postica*	Chraberce, Czechia	KY796487	KY796784	Western	HPCzech51					Yes	KY797024	KY797058	KY797093	MK190874	*w* Hypera1
HP6019	*Hypera postica*	Montana 1, USA	KY796430	KY796727	Western	HPUSAMO19	KY796961	HPIRANJ54	KY796259	HPUSAMO19	No					
HP6020	*Hypera postica*	Montana 1, USA	KY796431	KY796728	Western	HPUSAMO19					No					
HP6021	*Hypera postica*	Montana 1, USA	KY796432	KY796729	Western	HPUSAMO19			KY796260	HPUSAMO35	No					
HP6022	*Hypera postica*	Montana 1, USA	KY796433	KY796730	Western	HPUSAMO19			KY796261	HPUSAMO35	No					
HP6023	*Hypera postica*	Montana 1, USA	KY796434	KY796731	Western	HPUSAMO19					No					
HP6024	*Hypera postica*	Montana 1, USA	KY796435	KY796732	Western	HPUSAMO19					No					
HP6026	*Hypera postica*	Montana 1, USA	KY796436	KY796733	Western	HPUSAMO19					No					
HP6027	*Hypera postica*	Montana 1, USA	KY796437	KY796734	Western	HPUSAMO27					No					
HP6028	*Hypera postica*	Montana 1, USA	KY796438	KY796735	Western	HPUSAMO19					No					
HP6029	*Hypera postica*	Montana 1, USA	KY796439	KY796736	Western	HPUSAMO19					No					
HP6030	*Hypera postica*	Montana 1, USA	KY796440	KY796737	Western	HPUSAMO30					No					
HP6031	*Hypera postica*	Montana 1, USA	KY796441	KY796738	Western	HPUSAMO19					Yes	KY797025	KY797059	KY797094	MK190875	wHypera1
HP6032	*Hypera postica*	Montana 1, USA	KY796442	KY796739	Western	HPUSAMO19					No					
HP6033	*Hypera postica*	Montana 1, USA	KY796443	KY796740	Western	HPUSAMO19					Yes	KY797026	KY797060	KY797095	MK190876	*w* Hypera3
HP6034	*Hypera postica*	Montana 1, USA	KY796444	KY796741	Western	HPUSAMO19					Yes	KY797027	KY797061	KY797096	MK190877	*w* Hypera1
HP6035	*Hypera postica*	Montana 1, USA	KY796445	KY796742	Western	HPUSAMO19			KY796262	HPUSAMO35	Yes	KY797028	KY797062	KY797097	MK190878	*w* Hypera1
HP6036	*Hypera postica*	Montana 1, USA	KY796446	KY796743	Western	HPUSAMO36					No					
HP6037	*Hypera postica*	Montana 1, USA	KY796447	KY796744	Western	HPUSAMO19					No					
HP6038	*Hypera postica*	Montana 1, USA	KY796448	KY796745	Western	HPUSAMO19					No					
HP6039	*Hypera postica*	Montana 1, USA	KY796449	KY796746	Western	HPUSAMO19					No					
HP6041	*Hypera postica*	Montana 1, USA	KY796450	KY796747	Western	HPUSAMO19			KY796263	HPIRANJ56	Yes	KY797029	KY797063	KY797098	MK190879	*w* Hypera1
HP6042	*Hypera postica*	Montana 1, USA	KY796451	KY796748	Western	HPUSAMO19					No					
HP6043	*Hypera postica*	Montana 1, USA	KY796452	KY796749	Western	HPUSAMO19	KY796962	HPUSAMO43	KY796264	HPUSAMO35	No					
HP6044	*Hypera postica*	Montana 1, USA	KY796453	KY796750	Western	HPUSAMO19			KY796265	HPUSAMO44	No					
HP6047	*Hypera postica*	Montana 1, USA	KY796454	KY796751	Western	HPUSAMO19					No					
HP6048	*Hypera postica*	Montana 1, USA	KY796455	KY796752	Western	HPUSAMO48					No					
HP6051	*Hypera postica*	Montana 1, USA	KY796456	KY796753	Western	HPUSAMO51					No					
HP6054	*Hypera postica*	Montana 1, USA	KY796457	KY796754	Western	HPUSAMO19			KY796266	HPUSAMO54	No					
HP6056	*Hypera postica*	Montana 1, USA	KY796458	KY796755	Western	HPUSAMO19			KY796267	HPUSAMO56	Yes	KY797030	KY797064	KY797099	MK190880	*w* Hypera1
HP6058	*Hypera postica*	Montana 1, USA	KY796459	KY796756	Western	HPUSAMO19			KY796268	HPUSAMO58	No					
HP6059	*Hypera postica*	Montana 1, USA	KY796460	KY796757	Western	HPUSAMO19			KY796269	HPUSAMO35	Yes	KY797031	KY797065	KY797100	MK190881	*w* Hypera1
HP6060	*Hypera postica*	Montana 1, USA	KY796461	KY796758	Western	HPUSAMO19			KY796270	HPUSAMO60	Yes	KY797032	KY797066	KY797101	MK190882	*w* Hypera1

HP6070	*Hypera postica*	Montana 2, USA	KY796462	KY796759	Western	HPUSAMO19					No					
HP6071	*Hypera postica*	Montana 2, USA	KY796463	KY796760	Western	HPUSAMO19					No					
HP6072	*Hypera postica*	Montana 2, USA	KY796464	KY796761	Western	HPUSAMO19					No					
HP6075	*Hypera postica*	Montana 2, USA	KY796465	KY796762	Western	HPUSAMO19	KY796963	HPUSAMO43	KY796271	HPUSAMO35	Yes	KY797033	KY797067	KY797102	MK190883	*w*Hypera1
HP6076	*Hypera postica*	Montana 2, USA	KY796466	KY796763	Western	HPUSAMO19	KY796964	HPIRANJ54	KY796272	HPUSAMO35	Yes	KY797034	KY797068	KY797103	MK190884	*w*Hypera1
HP6077	*Hypera postica*	Montana 2, USA	KY796467	KY796764	Western	HPUSAMO19					No					
HP6078	*Hypera postica*	Montana 2, USA	KY796468	KY796765	Western	HPUSAMO19					No					
HP6080	*Hypera postica*	Montana 2, USA	KY796469	KY796766	Western	HPUSAMO19	KY796965	HPIRANJ54			Yes	KY797035	KY797069	KY797104	MK190885	*w*Hypera1
HP6081	*Hypera postica*	Montana 2, USA	KY796470	KY796767	Western	HPUSAMO19	KY796966	HPIRANJ54	KY796273	HPUSAMO35	Yes	KY797036	KY797070	KY797105	MK190886	*w*Hypera1
HP6082	*Hypera postica*	Montana 2, USA	KY796471	KY796768	Western	HPUSAMO19			KY796274	HPUSAMO35	Yes	KY797037	KY797071	KY797106	MK190887	*w*Hypera1
HP6083	*Hypera postica*	Montana 2, USA	KY796472	KY796769	Western	HPUSAMO19					No					
HP6085	*Hypera postica*	Montana 2, USA	KY796473	KY796770	Western	HPUSAMO85			KY796275	HPUSAMO35	Yes	KY797038	KY797072	KY797107	MK19088	*w*Hypera1
HP6087	*Hypera postica*	Montana 2, USA	KY796474	KY796771	Western	HPUSAMO19					Yes	KY797039	KY797073	KY797108	MK19089	*w*Hypera1
HP6088	*Hypera postica*	Montana 2, USA	KY796475	KY796772	Western	HPUSAMO88					No					
HP6089	*Hypera postica*	Montana 2, USA	KY796476	KY796773	Western	HPUSAMO19			KY796276	HPUSAMO35	Yes	KY797040	KY797074	KY797109	MK19090	*w*Hypera1
HP6090	*Hypera postica*	Montana 2, USA	KY796477	KY796774	Western	HPUSAMO19					Yes	KY797041	KY797075	KY797110	MK19091	*w*Hypera1
HP6093	*Hypera postica*	Montana 2, USA	KY796478	KY796775	Western	HPUSAMO19					Yes	KY797042	KY797076	KY797111	MK19092	*w*Hypera1
HP6094	*Hypera postica*	Montana 2, USA	KY796479	KY796776	Western	HPUSAMO19			KY796277	HPUSAMO35	No					
HP6095	*Hypera postica*	Montana 2, USA	KY796480	KY796777	Western	HPUSAMO19	KY796967	HPIRANJ54	KY796278	HPUSAMO35	Yes	KY797043	KY797077	KY797112	MK19093	*w*Hypera1
HP6099	*Hypera postica*	Montana 2, USA	KY796481	KY796778	Western	HPUSAMO19	KY796968	HPUSAMO99			No					
HP6100	*Hypera postica*	Montana 2, USA	KY796482	KY796779	Western	HPUSAMO19			KY796279	HPUSAMO35	No					
HP6188	*Hypera postica*	Toscana, Italy	KY796488	KY796785	Eastern	HPItaly88					No					
HP6189	*Hypera postica*	Toscana, Italy	KY796489	KY796786	Eastern	HPItaly89					No					
HP6190	*Hypera postica*	Toscana, Italy	KY796490	KY796787	Eastern	HPItaly90					No					
HP6191	*Hypera postica*	Taleghan, Iran	KY796426	KY796788	Eastern	HPIRANTA91					No					
HP6193	*Hypera postica*	Taleghan, Iran	KY796427	KY796723	Eastern	HPIRANTA93					No					
HP6194	*Hypera postica*	Taleghan, Iran	KY796428	KY796724	Eastern	HPIRANH93					No					
HP6197	*Hypera postica*	Taleghan, Iran	KY796429	KY796725	Western	HPIRANTA97					No					
HP6198	*Hypera postica*	Taleghan, Iran	KY796491	KY796726	Eastern	HPIRANTA98					No					
HP6258	*Hypera postica*	Missouri, USA	KY796492	KY796789	Eastern	HPUSAMI58			KY796280	HPUSAMO56	No					
HP6259	*Hypera postica*	Missouri, USA	KY796493	KY796790	Eastern	HPUSAMI58	KY796969	HPIRANH98			No					
HP6260	*Hypera postica*	Missouri, USA	KY796494	KY796791	Eastern	HPUSAMI58			KY796281	HPUSAMO56	No					
HP6261	*Hypera postica*	Missouri, USA	KY796495	KY796792	Eastern	HPUSAMI58			KY796282	HPUSAMO56	No					
HP6262	*Hypera postica*	Missouri, USA	KY796496	KY796793	Eastern	HPIRANH93	KY796970	HPUSAMI62	KY796283	HPUSAMO56	No					
HP6263	*Hypera postica*	Missouri, USA	KY796497	KY796794	Eastern	HPUSAMI58	KY796971	HPUSAMI62	KY796284	HPUSAMO56	No					
HP6264	*Hypera postica*	Missouri, USA	KY796498	KY796795	Eastern	HPIRANH93			KY796285	HPUSAMO56	No					
HP6265	*Hypera postica*	Missouri, USA	KY796499	KY796796	Eastern	HPUSAMI58			KY796286	HPUSAMO56	No					
HP6266	*Hypera postica*	Missouri, USA	KY796500	KY796797	Eastern	HPUSAMI58	KY796972	HPUSAMI66	KY796287	HPUSAMO56	No					
HP6267	*Hypera postica*	Missouri, USA	KY796501	KY796798	Eastern	HPUSAMI58					No					
HP6268	*Hypera postica*	Missouri, USA	KY796502	KY796799	Eastern	HPUSAMI58					No					
HP6269	*Hypera postica*	Missouri, USA	KY796503	KY796800	Eastern	HPUSAMI58			KY796288	HPUSAMO56	No					
HP6270	*Hypera postica*	Missouri, USA	KY796504	KY796801	Eastern	HPUSAMI58			KY796289	HPUSAMO56	No					
HP6271	*Hypera postica*	Missouri, USA	KY796505	KY796802	Eastern	HPUSAMI58			KY796290	HPUSAMO56	No					
HP6272	*Hypera postica*	Missouri, USA	KY796506	KY796803	Eastern	HPUSAMI58			KY796291	HPIRANJ56	No					
HP6273	*Hypera postica*	Missouri, USA	KY796507	KY796804	Eastern	HPUSAMI58					No					
HP6274	*Hypera postica*	Missouri, USA	KY796508	KY796805	Eastern	HPUSAMI58					No					
HP6275	*Hypera postica*	Missouri, USA	KY796509	KY796806	Eastern	HPUSAMI58	KY796973	HPUSAMO43	KY796292	HPUSAMO56	No					
HP6281	*Hypera postica*	Gwangju, Korea	KY796510	KY796807	Eastern	HPIRANT70	KY796974	HPKORCH81			Yes			KY797113		*w*Hypera1
HP6282	*Hypera postica*	Gwangju, Korea	KY796511	KY796808	Eastern	HPKORCH82	KY796975	HPKORCH82			No					
HP6283	*Hypera postica*	Gwangju, Korea	KY796512	KY796809	Western	HPKORCH83					No					
HP6284	*Hypera postica*	Gwangju, Korea	KY796513	KY796810	Eastern	HPIRANT70	KY796976	HPUSAMO43	KY796293	HPKORCH84	No					
HP6285	*Hypera postica*	Gwangju, Korea	KY796514	KY796811	Eastern	HPKORCH85	KY796977	HPIRANH98			Yes			KY797114		*w*Hypera1
HP6286	*Hypera postica*	Gwangju, Korea	KY796515	KY796812	Western	HPKORCH83					No					
HP6287	*Hypera postica*	Gwangju, Korea	KY796516	KY796813	Eastern	HPIRANT70	KY796978	HPIRANH98			No					
HP6288	*Hypera postica*	Gwangju, Korea	KY796517	KY796814	Eastern	HPIRANT70					No					
HP6289	*Hypera postica*	Gwangju, Korea	KY796518	KY796815	Western	HPUSAMO19	KY796979	HPIRANH98	KY796294	HPKORCH89	No					
HP6290	*Hypera postica*	Gwangju, Korea	KY796519	KY796816	Eastern	HPKORCH90	KY796980	HPUSAMO43			No					
HP6291	*Hypera postica*	Gwangju, Korea	KY796520	KY796817	Eastern	HPIRANT70	KY796981	HPIRANJ54			No					
HP6292	*Hypera postica*	Gwangju, Korea	KY796521	KY796818	Western	HPUSAMO19	KY796982	HPIRANH98			No					
HP6293	*Hypera postica*	Gwangju, Korea	KY796522	KY796819	Eastern	HPKORCH93	KY796983	HPIRANH98	KY796295	HPKORCH93	No					
HP6294	*Hypera postica*	Gwangju, Korea	KY796523	KY796820	Eastern	HPKORCH94			KY796296	HPKORCH94	No					

HP6295	*Hypera postica*	Gwangju, Korea	KY796524	KY796821	Eastern	HPKORCH82					No					
HP6296	*Hypera postica*	Gwangju, Korea	KY796525	KY796822	Eastern	HPKORCH94					No					
HP6297	*Hypera postica*	Gwangju, Korea	KY796526	KY796823	Eastern	HPIRANT70	KY796984	HPKORCH97			No					
HP6298	*Hypera postica*	Gwangju, Korea	KY796527	KY796824	Eastern	HPIRANT70					No					
HP6299	*Hypera postica*	Gwangju, Korea	KY796528	KY796825	Eastern	HPKORCH82	KY796985	HPUSAMO43	KY796297	HPIRANJ56	Yes	KY797044	KY797078	KY797115	MK19094	*w* Hypera1
HP6300	*Hypera postica*	Gwangju, Korea	KY796529	KY796826	Eastern	HPIRANT70	KY796986	HPIRANH98	KY796298	HPIRANJ56	Yes			KY797116		*w* Hypera1
HP6301	*Hypera postica*	Gwangju, Korea	KY796530	KY796827	Eastern	HPIRANT70					No					
HP6334	*Hypera postica*	Nebraska, USA	KY796531	KY796828	Western	HPUSAMO19					No					
HP6335	*Hypera postica*	Nebraska, USA	KY796532	KY796829	Western	HPUSAMO19					No					
HP6336	*Hypera postica*	Nebraska, USA	KY796533	KY796830	Western	HPUSAMO19					No					
HP6337	*Hypera postica*	Nebraska, USA	KY796534	KY796831	Western	HPUSAMO19					No					
HP6338	*Hypera postica*	Nebraska, USA	KY796535	KY796832	Western	HPUSAMO19					No					
HP6339	*Hypera postica*	Nebraska, USA	KY796536	KY796833	Western	HPUSAMO19					No					
HP6340	*Hypera postica*	Nebraska, USA	KY796537	KY796834	Western	HPUSAMO19					No					
HP6341	*Hypera postica*	Nebraska, USA	KY796538	KY796835	Western	HPUSAMO19					No					
HP6342	*Hypera postica*	Nebraska, USA	KY796539	KY796836	Western	HPUSAMO19					No					
HP6343	*Hypera postica*	Nebraska, USA	KY796540	KY796837	Western	HPUSAMO19					No					
HP6344	*Hypera postica*	Nebraska, USA	KY796541	KY796838	Western	HPUSAMO19					No					
HP6345	*Hypera postica*	Nebraska, USA	KY796542	KY796839	Western	HPUSANE45					No					
HP6346	*Hypera postica*	Nebraska, USA	KY796543	KY796840	Western	HPUSAMO19					No					
HP6347	*Hypera postica*	Nebraska, USA	KY796544	KY796841	Western	HPUSAMO19					No					
HP6348	*Hypera postica*	Nebraska, USA	KY796545	KY796842	Western	HPUSAMO19					Yes	KY797045	KY797079	KY797117	MK19095	*w* Hypera1
HP6349	*Hypera postica*	Nebraska, USA	KY796546	KY796843	Western	HPUSANE4					No					
HP6350	*Hypera postica*	Nebraska, USA	KY796547	KY796844	Western	HPUSAMO19					No					
HP6351	*Hypera postica*	Nebraska, USA	KY796548	KY796845	Western	HPUSAMO19					No					
HP6352	*Hypera postica*	Nebraska, USA	KY796549	KY796846	Western	HPUSAMO19	KY796987	HPUSANE52	KY796299	HPUSANE52	No					
HP6353	*Hypera postica*	Nebraska, USA	KY796550	KY796847	Western	HPUSAMO19					No					
HP6354	*Hypera postica*	Nebraska, USA	KY796551	KY796848	Western	HPUSANE4					No					
HP6355	*Hypera postica*	Nebraska, USA	KY796552	KY796849	Western	HPUSAMO19					No					
HP6356	*Hypera postica*	Nebraska, USA	KY796553	KY796850	Western	HPUSAMO19	KY796988	HPUSAMO43			Yes	KY797046	KY797080	KY797118	MK19096	*w* Hypera1
HP6357	*Hypera postica*	Nebraska, USA	KY796554	KY796851	Western	HPUSAMO19	KY796989	HPUSANE57	KY796300	HPUSANE57	No					
HP6358	*Hypera postica*	Nebraska, USA	KY796555	KY796852	Western	HPUSAMO19	KY796990	HPUSAMI66	KY796301	HPUSANE56	No					
HP6359	*Hypera postica*	Nebraska, USA	KY796556	KY796853	Western	HPUSAMO19					No					
HP6360	*Hypera postica*	Nebraska, USA	KY796557	KY796854	Western	HPUSAMO19					No					
HP6361	*Hypera postica*	Nebraska, USA	KY796558	KY796855	Western	HPUSAMO19					No					
HP6437	*Hypera postica*	California, USA	KY796559	KY796856	Western	HPUSAMO19			KY796302	HPUSAMO35	No					
HP6438	*Hypera postica*	California, USA	KY796560	KY796857	Western	HPUSAMO19					No					
HP6439	*Hypera postica*	California, USA	KY796561	KY796858	Western	HPUSAMO19			KY796303	HPUSAMO35	Yes	KY797047	KY797081	KY797119	MK19097	*w* Hypera1
HP6440	*Hypera postica*	California, USA	KY796562	KY796859	Western	HPUSAMO19			KY796304	HPUSACA40	No					
HP6441	*Hypera postica*	California, USA	KY796563	KY796860	Western	HPUSAMO19			KY796305	HPUSAMO35	No					
HP6442	*Hypera postica*	California, USA	KY796564	KY796861	Western	HPUSAMO19	KY796991	HPUSACA42			No					
HP6443	*Hypera postica*	California, USA	KY796565	KY796862	Western	HPUSAMO19	KY796992	HPUSAMO43			Yes	KY797048	KY797082	KY797120	MK19098	*w* Hypera1
HP6444	*Hypera postica*	California, USA	KY796566	KY796863	Western	HPUSAMO19					No					
HP6445	*Hypera postica*	California, USA	KY796567	KY796864	Western	HPUSAMO19			KY796306	HPUSAMO35	No					
HP6446	*Hypera postica*	California, USA	KY796568	KY796865	Western	HPUSAMO19			KY796307	HPUSACA46	No					
HP6447	*Hypera postica*	California, USA	KY796569	KY796866	Western	HPUSAMO19	KY796993	HPUSAMO43			Yes	KY797049	KY797083	KY797121	MK19099	*w* Hypera1
HP6448	*Hypera postica*	California, USA	KY796570	KY796867	Western	HPUSAMO19	KY796994	HPUSAMO43	KY796308	HPUSAMO35	Yes	KY797050	KY797084	KY797122	MK19100	*w* Hypera1
HP6450	*Hypera postica*	California, USA	KY796571	KY796868	Western	HPUSACA50	KY796995	HPUSACA42	KY796310	HPUSAMO35	No					
HP6452	*Hypera postica*	California, USA	KY796572	KY796869	Western	HPUSAMO19	KY796996		KY796311	HPUSAMO35	No					
HP6453	*Hypera postica*	California, USA	KY796573	KY796870	Western	HPUSAMO19					Yes	KY797051	KY797085	KY797123	MK19101	*w* Hypera1
HP6454	*Hypera postica*	California, USA	KY796574	KY796871	Western	HPUSAMO19			KY796312	HPUSAMO35	No					
HP6455	*Hypera postica*	California, USA	KY796575	KY796872	Western	HPUSAMO19			KY796313	HPUSAMO35	Yes	KY797052	KY797086	KY797124	MK19102	*w* Hypera2
HP6456	*Hypera postica*	California, USA	KY796576	KY796873	Western	HPUSAMO19	KY796997	HPUSAMO43			No					
HP6457	*Hypera postica*	California, USA	KY796577	KY796874	Western	HPUSAMO19			KY796314	HPUSAMO35	No					
HP6458	*Hypera postica*	California, USA	KY796578	KY796875	Western	HPUSAMO19	KY796998	HPUSAMO43	KY796315	HPUSAMO35	No					
HP6459	*Hypera postica*	California, USA	KY796579	KY796876	Western	HPUSAMO19					No					
HP6460	*Hypera postica*	California, USA	KY796580	KY796877	Western	HPUSAMO19	KY796999	HPUSACA42			No					
HP6513	*Hypera postica*	Plovdic, Bulgaria	KY796581	KY796878	Eastern	HPIRANH93			KY796316	HPBULPL13	No					
HP6514	*Hypera postica*	Plovdic, Bulgaria	KY796582	KY796879	Eastern	HPBULPL14	KY797000	HPBULPL14	KY796317	HPBULPL14	Yes			KY797125		*w* Hypera1
HP6515	*Hypera postica*	Plovdic, Bulgaria	KY796583	KY796880	Eastern	HPBULPL15					No					
HP6516	*Hypera postica*	Plovdic, Bulgaria	KY796584	KY796881	Eastern	HPBULPL16	KY797001	HPBULPL16			No					
HP6517	*Hypera postica*	Plovdic, Bulgaria	KY796585	KY796882	Eastern	HPBULPL17	KY797002	HPBULPL17			Yes	KY797053	KY797087	KY797126	MK19103	*w* Hypera1

HP6518	*Hypera postica*	Plovdic, Bulgaria	KY796586	KY796883	Eastern	HPBULPL18	KY797003	HPIRANJ54	KY796318	HPBULPL18	Yes			KY797127		*w* Hypera1
HP6519	*Hypera postica*	Plovdic, Bulgaria	KY796587	KY796884	Eastern	HPBULPL19					No					
HP6520	*Hypera postica*	Plovdic, Bulgaria	KY796588	KY796885	Eastern	HPBULPL20	KY797004	HPBULPL20			Yes			KY797128		*w* Hypera1
HP6521	*Hypera postica*	Plovdic, Bulgaria	KY796589	KY796886	Eastern	HPBULPL21					No					
HP6522	*Hypera postica*	Plovdic, Bulgaria	KY796590	KY796887	Eastern	HPBULPL22					No					
HP6523	*Hypera postica*	Plovdic, Bulgaria	KY796591	KY796888	Eastern	HPBULPL23			KY796319	HPBULPL23	No					
HP6524	*Hypera postica*	Plovdic, Bulgaria	KY796592	KY796889	Eastern	HPBULPL24	KY797005	HPBULPL24			No					
HP6525	*Hypera postica*	Plovdic, Bulgaria	KY796593	KY796890	Eastern	HPBULPL25			KY796320	HPKORCH94	No					
HP6526	*Hypera postica*	Plovdic, Bulgaria	KY796594	KY796891	Eastern	HPIRANH93					Yes			KY797129		*w* Hypera1
HP6527	*Hypera postica*	Plovdic, Bulgaria	KY796595	KY796892	Eastern	HPBULPL27					No					
HP6528	*Hypera postica*	Plovdic, Bulgaria	KY796596	KY796893	Eastern	HPBULPL28					No					
HP6529	*Hypera postica*	Plovdic, Bulgaria	KY796597	KY796894	Eastern	HPBULPL29					No					
HP6530	*Hypera postica*	Plovdic, Bulgaria	KY796598	KY796895	Eastern	HPIRANH93					No					
HP6531	*Hypera postica*	Plovdic, Bulgaria	KY796599	KY796896	Eastern	HPBULPL27	KY797006	HPBULPL31	KY796321	HPBULPL31	No					
HP6532	*Hypera postica*	Plovdic, Bulgaria	KY796600	KY796897	Eastern	HPIRANH93			KY796322	HPIRANJ57	No					
HP6533	*Hypera postica*	Plovdic, Bulgaria	KY796601	KY796898	Eastern	HPBULPL33			KY796323	HPBULPL33	No					
HP6534	*Hypera postica*	Plovdic, Bulgaria	KY796602	KY796899	Eastern	HPBULPL34					No					
HP6535	*Hypera postica*	Plovdic, Bulgaria	KY796603	KY796900	Eastern	HPIRANH93			KY796324	HPBULPL35	No					
HP6536	*Hypera postica*	Plovdic, Bulgaria	KY796604	KY796901	Eastern	HPBULPL36	KY797007	HPIRANJ54	KY796325	HPBULPL36	Yes			KY797130		*w* Hypera1
HP6545	*Hypera postica*	Okinawa, Japan	KY796605	KY796902	Eastern	HPJAPOK45	KY797008	HPIRANH98	KY796326	HPJAPOK45	No					
HP6546	*Hypera postica*	Okinawa, Japan	KY796606	KY796903	Eastern	HPIRANT70					No					
HP6547	*Hypera postica*	Okinawa, Japan	KY796607	KY796904	Western	HPUSAMO19					No					
HP6548	*Hypera postica*	Okinawa, Japan	KY796608	KY796905	Eastern	HPIRANT70					Yes	KY797054	KY797088	KY797131	MK19104	*w* Hypera1
HP6549	*Hypera postica*	Okinawa, Japan	KY796609	KY796906	Eastern	HPIRANT70	KY797009	HPIRANH98	KY796327	HPJAPOK49	No			KY797132		*w* Hypera1
HP6550	*Hypera postica*	Okinawa, Japan	KY796610	KY796907	Western	HPUSAMO19			KY796328	HPUSAMO44	No					
HP6551	*Hypera postica*	Okinawa, Japan	KY796611	KY796908	Eastern	HPIRANT70					Yes			KY797133		*w* Hypera1
HP6552	*Hypera postica*	Okinawa, Japan	KY796612	KY796909	Eastern	HPKORCH82					Yes			KY797134		*w* Hypera1
HP6553	*Hypera postica*	Okinawa, Japan	KY796613	KY796910	Eastern	HPJAPOK53			KY796329	HPJAPOK53	Yes			KY797135		*w* Hypera1
HP6554	*Hypera postica*	Okinawa, Japan	KY796614	KY796911	Eastern	HPIRANT70	KY797010	HPIRANJ54	KY796330	HPJAPOK54	Yes			KY797136		*w* Hypera1
HP6555	*Hypera postica*	Okinawa, Japan	KY796615	KY796912	Eastern	HPJAPOK55			KY796331	HPJAPOK53	No					
HP6556	*Hypera postica*	Okinawa, Japan	KY796616	KY796913	Western	HPUSAMO19					No					
HP6557	*Hypera postica*	Okinawa, Japan	KY796617	KY796914	Eastern	HPIRANT70			KY796332	HPJAPOK57	Yes			KY797137		*w* Hypera1
HP6558	*Hypera postica*	Okinawa, Japan	KY796618	KY796915	Western	HPJAPOK58	KY797011	HPJAPOK58			No					
HP6559	*Hypera postica*	Okinawa, Japan	KY796619	KY796916	Western	HPUSAMO19					Yes			KY797138		*w* Hypera1
HP6560	*Hypera postica*	Okinawa, Japan	KY796620	KY796917	Eastern	HPKORCH82			KY796333	HPIRANJ56	No					
HP6561	*Hypera postica*	Okinawa, Japan	KY796621	KY796918	Western	HPUSAMO19	KY797012	HPJAPOK61	KY796334	HPJAPOK61	Yes			KY797139		*w* Hypera1
HP6562	*Hypera postica*	Okinawa, Japan	KY796622	KY796919	Western	HPUSAMO19	KY797013	HPJAPOK58	KY796335	HPJAPOK62	Yes			KY797140		*w* Hypera1
HP6563	*Hypera postica*	Okinawa, Japan	KY796623	KY796920	Western	HPUSAMO19					No					
HP6564	*Hypera postica*	Okinawa, Japan	KY796624	KY796921	Western	HPUSAMO19	KY797014	HPJAPOK64	KY796336	HPIRANJ56	Yes			KY797141		*w* Hypera1
HP6572	*Hypera postica*	Knezha, Bulgaria	KY796625	KY796922	Eastern	HPBULKN72	KY797015	HPUSAMI62	KY796337	HPBULPL14	No					
HP6574	*Hypera postica*	Knezha, Bulgaria	KY796626	KY796923	Eastern	HPBULKN74					No					
HP6576	*Hypera postica*	Knezha, Bulgaria	KY796627	KY796924	Eastern	HPBULKN76	KY797016	HPBULKN76			No					
HP6577	*Hypera postica*	Knezha, Bulgaria	KY796628	KY796925	Eastern	HPIRANH93					No					
HP6578	*Hypera postica*	Knezha, Bulgaria	KY796629	KY796926	Eastern	HPBULKN78					No					
HP6579	*Hypera postica*	Knezha, Bulgaria	KY796630	KY796927	Eastern	HPBULKN79					No					
HP6580	*Hypera postica*	Knezha, Bulgaria	KY796631	KY796928	Eastern	HPBULKN80					No					
HP6581	*Hypera postica*	Knezha, Bulgaria	KY796632	KY796929	Eastern	HPBULKN81					No					
HP6582	*Hypera postica*	Knezha, Bulgaria	KY796633	KY796930	Western	HPBULKN82					No					
HP6583	*Hypera postica*	Knezha, Bulgaria	KY796634	KY796931	Eastern	HPBULPL20					No					
HP6584	*Hypera postica*	Knezha, Bulgaria	KY796635	KY796932	Western	HPBULKN84			KY796339	HPBULKN84	No					
HP6585	*Hypera postica*	Knezha, Bulgaria	KY796636	KY796933	Eastern	HPBULKN85					No					
HP6586	*Hypera postica*	Knezha, Bulgaria	KY796637	KY796934	Eastern	HPBULKN86			KY796340	HPBULKN86	Yes			KY797142		*w* Hypera1
HP6587	*Hypera postica*	Knezha, Bulgaria	KY796638	KY796935	Eastern	HPBULKN87			KY796341	HPBULKN87	No					
HP6588	*Hypera postica*	Knezha, Bulgaria	KY796639	KY796936	Eastern	HPIRANH93					No					
HP6589	*Hypera postica*	Knezha, Bulgaria	KY796640	KY796937	Eastern	HPBULKN74			KY796342	HPBULKN89	No					
HP6591	*Hypera postica*	Lozitsa, Bulgaria	KY796642	Yes	Western	HPBULLO91	KY797017		KY796344		Yes			KY797143		*w* Hypera1
HV6592	*Hypera viciae*	Knezha, Bulgaria	No	KY796939		No	KY797018	H. viciae2	KY796345	H. viciae2	No					
HV6593	*Hypera viciae*	Knezha, Bulgaria	KY796643	KY796940		H. viciae3	KY797019	H. viciae3	KY796346	H. viciae3	Yes			KY797144		*w* Hypera1
HV6594	*Hypera viciae*	Knezha, Bulgaria	KY796644	KY796941		H. viciae4	KY797020	H. viciae4	KY796347	H. viciae4	Yes			KY797145		*w* Hypera1
HV6595	*Hypera viciae*	Knezha, Bulgaria	KY796645	No		H. viciae5			KY796348	H. viciae2	Yes			KY797146		*w* Hypera1
