# Supplementary material for: Global genetic diversity, lineage distribution, and Wolbachia infection of the alfalfa weevil Hypera postica (Coleoptera: Curculionidae)
Source: Ecol Evol. 2019 Aug 6;9(17):9546–63. doi: 10.1002/ece3.5474 (PMC6745856; doi:10.1002/ece3.5474)
Supplement: Supplementary file 2 [file ECE3-9-9546-s002.docx]

**Designing primer**

Historically, part of COI, COII, and CytB was used for lineage determination of American and Japanese populations of *H. postica* by adopting two pairs of common Coleoptera developed primers (Erney et al., 1996; Hsaio, 1996). The fragment contained the end of CytB, tRNA^ser,^ and ND1 is too short (300 bp length) and the second primer contained the end of COI, tRNA^Leu,^ and COII is too long (881 bp) (Kuwata et al., 2005) which can cause some problems in both amplification and sequencing. Therefore, we selected the barcode region in Cytochrome Oxidase subunit I (COI) and Cytochrome Oxidase subunit B (CytB) as two well-known mitochondrial genes for population genetic and phylogenetic analysis.

Böttger et al. (2013) amplified several nuclear genes in order to find a distinct divergence pattern among lineages. While they failed to amplified Topoisomerase I (TI) and Cinnamyl Alcohol Dehydrogenease (CAD), few sequences of Ribosomal RNA (18s rRNA and ITS), Elongation Factor-1 alpha (EF1a), Arginine Kinase (AK) and alpha Spectrin genes were obtained by using general primers (Böttger et al., 2013). According to the result of the mentioned study, the uniformity was observed in 18s and AK and unsortable variation was detected in other nuclear genes. Based on previous investigations, we used EF1a as a powerful nuclear marker in weevils that can distinguish sibling species (Jordal, 2002; Mayer et al., 2015; Toševski et al., 2015) and CAD as an informative gene in phylogenetic study (Jordal, 2015; Jordal et al., 2011; Wild and Maddison, 2008). We also tried several Wingless (Wg) primers (Wild and Maddison, 2008) for a few samples but the sequencing was failed. In addition, we borrowed some newly developed primers for weevils such as Polyadenylate Binding Protein (PyA) and Ubiquitin-like modifier activating enzyme 5 (UBA5) (Pistone et al., 2016). The UBA5 was not amplified and the preliminary result of PyA sequences showed high uniformity and only one non-informative mutation among the eight samples. Therefore, in this study EF1a and CAD were used as the final nuclear markers.

Mitochondrial and nuclear Fragments were amplified using specific primers designed in this study (Sup. TableS1). At first, a longer fragment of each gene was amplified by primitive designed or commonly developed primers. In the next step, few sequences of our samples (from different populations) resulted from primitive primers were used for designing final primers. For mitochondrial genes, a semi-completed mitochondrial genome of *Hypera postica* (Genbank accession: JN163953; (Haran et al., 2013) was adopted as a first template. To design EF1a primer, sequences of *H. postica* from Böttger et al. (2013) were used as a template. As there was no previous sequences of CAD for *H. postica* in GenBank, the sequences of other Curculionidae species (from 800 – 440 bp) were aligned and used (Jordal et al., 2011).

**PCR Conditions**

The AccuPower PreMix (Bioneer, Korea) was used for all PCRs (mitochondrial, nuclear and *Wolbachia* genes). The cycling profile for PCR of mitochondrial and nuclear genes was: 95°C for 4 min, 30 cycles of 95°C for 1 min, 48°C (for mitochondrial genes) or 55°C (for nuclear genes) for 1 min, 72°C for 1 min and a final extension period of 70°C for 10 min. PCR products were purified using Accu-Prep® PCR Purification Kit reagents (Bioneer, Korea). DNA Sanger sequencing was conducted using the ABI PRISM® BigDye® Terminator v. 3.1 Cycle Sequencing Kit with an ABI 3100 Genetic Analyzer (Macrogene, South Korea). All products were sequenced from both directions.

**References**:

Böttger, J.A.A., Bundy, C.S., Oesterle, N., Hanson, S.F., 2013. Phylogenetic analysis of the alfalfa weevil complex (Coleoptera: Curculionidae) in North America. Journal of Economic Entomology 106, 426-436.

Erney, S., Pruess, K., Danielson, S., Powers, T., 1996. Molecular differentiation of alfalfa weevil strains (Coleoptera: Curculionidae). Annals of the Entomological Society of America 89, 804-811.

Haran, J., Timmermans, M.J., Vogler, A.P., 2013. Mitogenome sequences stabilize the phylogenetics of weevils (Curculionoidea) and establish the monophyly of larval ectophagy. Molecular Phylogenetics and Evolution 67, 156-166.

Hsaio, T.H., 1996. Studies of interactions between alfalfa weevil strains, Wolbachia endosymbionts and parasitoids. In: Symondson, W.O.C., Liddel, J.E. (Eds.), The ecology of agriculture pests. Chapman & Hall, London.

Jordal, B., 2002. Elongation Factor 1 α resolves the monophyly of the haplodiploid ambrosia beetles Xyleborini (Coleoptera: Curculionidae). Insect molecular biology 11, 453-465.

Jordal, B.H., 2015. Molecular phylogeny and biogeography of the weevil subfamily Platypodinae reveals evolutionarily conserved range patterns. Molecular phylogenetics and evolution 92, 294-307.

Jordal, B.H., Sequeira, A.S., Cognato, A.I., 2011. The age and phylogeny of wood boring weevils and the origin of subsociality. Molecular Phylogenetics and Evolution 59, 708-724.

Kuwata, R., Tokuda, M., Yamaguchi, D., Yukawa, J., 2005. Coexistence of two mitochondrial DNA haplotypes in Japanese populations of *Hypera postica* (Col., Curculionidae). Journal of Applied Entomology 129, 191-197.

Mayer, F., Piel, F.B., Cassel‐Lundhagen, A., Kirichenko, N., Grumiau, L., Økland, B., Bertheau, C., Grégoire, J.C., Mardulyn, P., 2015. Comparative multilocus phylogeography of two Palaearctic spruce bark beetles: influence of contrasting ecological strategies on genetic variation. Molecular ecology 24, 1292-1310.

Pistone, D., Mugu, S., Jordal, B.H., 2016. Genomic Mining of Phylogenetically Informative Nuclear Markers in Bark and Ambrosia Beetles. PloS one 11, e0163529.

Toševski, I., Caldara, R., Jović, J., Hernández‐Vera, G., Baviera, C., Gassmann, A., Emerson, B.C., 2015. Host‐associated genetic divergence and taxonomy in the Rhinusa pilosa Gyllenhal species complex: an integrative approach. Systematic Entomology 40, 268-287.

Wild, A.L., Maddison, D.R., 2008. Evaluating nuclear protein-coding genes for phylogenetic utility in beetles. Molecular phylogenetics and evolution 48, 877-891.
