# Supplementary material for: Global genetic diversity, lineage distribution, and Wolbachia infection of the alfalfa weevil Hypera postica (Coleoptera: Curculionidae)
Source: Ecol Evol. 2019 Aug 6;9(17):9546–63. doi: 10.1002/ece3.5474 (PMC6745856; doi:10.1002/ece3.5474)
Supplement: Supplementary file 3 [file ECE3-9-9546-s003.docx]

**A deeper look at mitochondrial divergence among two lineages**

The result of the obtained mitochondrial genes (1776 bp of COI and CytB sequences) revealed a deep divergence between two previously defined clades (average of 90 nucleotide differences, 6.15%). with the range between 5.6 – 6.7%, 33 fixed differences, 180 unique mutations polymorphic in Eastern lineage compare to 37 unique mutations polymorphic in Western lineage and only 21 shared mutations) (Sup. Table S3). Some part of Mt DNA of Eastern lineage carries the highest diversity range. There, the highest nucleotide diversity was observed in the following gene position; 638-693bp of COI, 41-100bp of CytB and 221-280bp of CytB with respectively 4.032%, 3.291%, and 3.176% mean nucleotide differences and the most three conserved locations were at 1-59bp, 518-569bp and 278-337bp of COI with respectively 0.04%, 0.119%, and 0.156% mean nucleotide differences. In the Western lineage, the highest diversity was detected at 327-386pb and 298-357bp of COI and 741-783bp of CytB with respectively 0.049%, 0.439% and 0.397% nucleotide differences and four places in the genes; 378-437pb of COI and 621-680bp, 301-360bp, 141-240bp, 21-80bp of Cytb was totally conserved with zero nucleotide diversity. To monitor the nucleotide diversity changes in the length of mitochondrial genes of each lineage and their nucleotide differences please refer to Sup. Fig. S1.
