# Supplementary material for: Global genetic diversity, lineage distribution, and Wolbachia infection of the alfalfa weevil Hypera postica (Coleoptera: Curculionidae)
Source: Ecol Evol. 2019 Aug 6;9(17):9546–63. doi: 10.1002/ece3.5474 (PMC6745856; doi:10.1002/ece3.5474)

Supp. Table S1: List of designed primers which were used for PCR amplification and sequencing in this study.

| Region | Primer name | Direction | Sequences (5`-3`) | Size |
| --- | --- | --- | --- | --- |
| COI (barcode) | HPBarcodF1 | F | TCAGCCATTTTACCGAAC | 693bp |
|  | HPBarcodR2 | R | CTTCAGGGTGTCCAAAAAATC |  |
| CytB | HPCytBF1 | F | ATTGTCCAAACATAGAATTAGC | 783bp |
|  | HPCytBR2 | R | GATAGAAAATATTCTTCAAAATA |  |
| Ef1a | HPEFlF2 | F | CGCCAAATATTATGTCACCATCA | 806bp |
|  | HPEFlR2 | R | GCAATTCCTTGACTGAAACG |  |
| CAD | HPCADF2 | F | GACAACTGCATWACAGTTTG | 402bp |
|  | HPCADR2 | R | GCTTCCAATCTTGGTACTCACTCGA |  |

Supp. Table S2: Detailed information of adopted sequences from MLST *Wolbachia* database.

| Registered ID | gatB, coxA, fbpA | Strain name | Reported Host | Host’s Order | Supergroup |
| --- | --- | --- | --- | --- | --- |
| 502 | 9, 180, 248 | - | - | - | B |
| 326 | 139, 125, 200 | - | - | - | B |
| 32 | 9, 9, 10 | Osca_B | *Ostrinia scapulalis* | Lepidoptera | B |
| 19 | 9, 14,14 | Calt_B | *Chelymorpha alternans* | Coleoptera | B |
| 23 | 12, 12, 22 | Aepo_B | *Acraea eponina* | Lepidoptera | B |
| 25 | 9, 25, 25 | Ttai_B | *Liriomyza rifoliita* | Diptera | B |
| 40 | 4, 14, 4 | Hbol_B_wBol1 | *Hypolimnas bolina B* | Lepidoptera | B |
| 73 | 108, 73, 9 | Lmel_B | *Lycaeides melissa* | Lepidoptera | B |
| 99 | 12, 14, 41 | Hony_B | *Horaga onyx* | Lepidoptera | B |
| 132 | 70, 64, 4 | Tthe_B | *Thersamonia thersamon* | Lepidoptera | B |
| 267 | 109, 76, 27 | Dcit_B_wDc01 | *Diaphorina citri* | Hemiptera | B |
| 296 | 9, 105, 180 | Lory_B | *Lissorhoptrus oryzophilus* | Coleoptera | B |
| 297 | 39, 14, 4 | Ehec_B_CI | *Eureman hecabe* | Lepidoptera | B |
| 461 | 9, 9, 10 | Eaet_1 | *Erebia aethiops* | Lepidoptera | B |
| 1820 | 253, 235, 9 | wPhar | *Petrobia harti* | Tetranychidae | B |
| 283 | 125, 4, 6 | wAli_B | *Diaphorencyrtus aligarhensis* | Prostigmata | B |
| 69 | 9, 9, 10 | Pdom_B_JKS1 | *Polistes dominulus* | Hymenoptera | B |
| 210 | 16,14,4 | Cama_B_wAma | *Colotis amata* | Lepidoptera | B |
| 450 | 9, 14, 9 | Apun_B | *Amblyptilia punctidactyla* | Lepidoptera | B |
| 1684 | 235, 227, 9 | Hhor_B_wHho | *Hyposoter horticola* | Hymenoptera | B |
| 1 | 1, 1, 1 | Dmel_A_wMel | *Drosophila melanogaster* | Diptera | A |
| 38 | 67, 6, 8 | Hbol_A_wBol2 | *Hypolimnas bolina* A | Lepidoptera | A |
| 55 | 8, 84, 160 | wCer1_A | *Rhagoletis cerasi* | Diptera | A |
| 68 | 32, 33, 37 | Aape_A_CDP21 | *Agelenopsis aperta* | Araneae | A |
| 96 | 54, 52, 62 | wAlu | *Aganaspis alujai* | Hymenoptera | A |
| 98 | 37, 37, 40 | Jale_A | *Jamides alecto* | Lepidoptera | A |

Supp. Table S3: Divergence between two lineages in the integrated zone. N: Number of fixed differences, ME: Mutations polymorphic in Eastern lineage specimens, but monomorphic in Western lineage specimens, MW: Mutations polymorphic in Western lineage specimens, but monomorphic in Eastern lineage specimens, SM: Shared mutations, AN: Average number of nucleotide differences between two lineages, Dxy: Average number of nucleotide subs. per site between lineages, Da: Number of network nucleotide subs. per site between lineages. There was only one Western lineage sample among the Taleghan population, therefore, SD could not be calculated.

| Population | N | ME | MW | SM | AN | Dxy | Da |
| --- | --- | --- | --- | --- | --- | --- | --- |
| Iran-Taleghan | 70 | 53 | 0 | 0 | 91.400 | 0.06192 | 0.05369 |
| Korea | 80 | 22 | 2 | 1 | 89.926 | 0.06093 ± 0.01961 | 0.05926 ± 0.01962 |
| Japan | 79 | 20 | 1 | 0 | 89.657 | 0.06074 ± 0.02012 | 0.05930 ± 0.02014 |
| Bulgaria Knezha | 64 | 58 | 5 | 1 | 89.500 | 0.06064 ± 0.01557 | 0.05363 ± 0.1566 |
| All populations | 33 | 180 | 37 | 21 | 90.772 | 0.06150 ± 0.00409 | 0.05480 ± 0.00411 |

Supp. Table S4. List of primers which were used to detect *Wolbachia* in this study mainly from MLST([Baldo et al., 2006b](#_ENREF_6)) and ARM primer ([Schneider et al., 2014](#_ENREF_95)). The mentioned size is approximate in some genes.

| Gene | Product | Direction | Sequences (5`-3`) | Size |
| --- | --- | --- | --- | --- |
| gatB | Gln Amidotransferase, subunit B | gatB_F1 | GAKTTAAAYCGYGCAGGBGTT | 429bp |
|  |  | gatB_R1 | TGGYAAYTCRGGYAAAGATGA |  |
| coxA | Cytochrome c oxidase, subunit I | coxA_F1 | TTGGRGCRATYAACTTTATAG | 445bp |
|  |  | coxA_R1 | CTAAAGACTTTKACRCCAGT |  |
| hcpA | Conserved hypothetical protein | hcpA_F1 | GAAATARCAGTTGCTGCAAA | 434bp |
|  |  | hcpA_R1 | GAAAGTYRAGCAAGYTCTG |  |
| ftsZ | Cell division protein | ftsZ_F1 | ATYATGGARCATATAAARGATAG | 435bp |
|  |  | ftsZ_R1 | CRAGYAATGGATTRGATAT |  |
| fbpA | Fructose-bisphosphate aldolase | fbpA_F1 | GCTGCTCCRCTTGGYWTGAT | 461bp |
|  |  | fbpA_R1 | CCRCCAGARAAAAYYACTATTC |  |
| ARM | A-Supergroup repeat motif | ARMF | TTCGCCAATCTGCAGATTAAA | 315bp |
|  |  | ARMR | TTGTCAAGCGTTTAAAAC |  |

Supp. Table S5: The result of HKA and McDonald–Kreitman test for mitochondrial genes. None of the values were significant. W: Western, E: Eastern, N: Native Eastern populations (Including Iranian, Italian and Bulgaria), I: Infected, U: Uninfected

|  | HKA Test | | McDonald–Kreitman test | | |
| --- | --- | --- | --- | --- | --- |
| Comparison groups | X-square | P value | Neutrality Index | Alfa value | Fisher's exact test. P-value |
| W vs E | 0.2196 | 0.2196 | 2.453 | -1.453 | 0.053 |
| E.N.I vs E.U | 0.005 | 0.9429 | 1.631 | -1.011 | 0.236 |
| E.N.I vs E.N.U | 0.008 | 0.9284 | 1.571 | -1.231 | 0.191 |

Supp. Fig. S1. Nucleotide diversity in each lineage and an average number of these differences between lineages in the mitochondrial genes. Position 1-784 is CytB and 785 to the end is COI. Pi1: Nucleotide diversity in Eastern lineage, Pi2: Nucleotide diversity in Western lineage and Dxy: Average number of nucleotide subs. per site between lineages


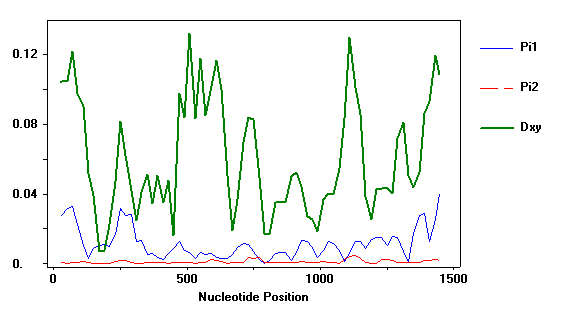


Supp. Fig. S2 **Bayesian tree based on concatenated CytB and COI haplotype sequences**. Each node number represents the posterior probability value. The blue line is an indicator for Eastern lineage, blue line for Western lineage, green line for outgroups, and black circle is posterior probability value, which is above 95%.

Supp. Fig. S2 **Bayesian tree based on concatenated CytB and COI haplotype sequences**. Each node number represents the posterior probability value. The blue line is an indicator for Eastern lineage, blue line for Western lineage, green line for outgroups, and black circle is posterior probability value, which is above 95%.


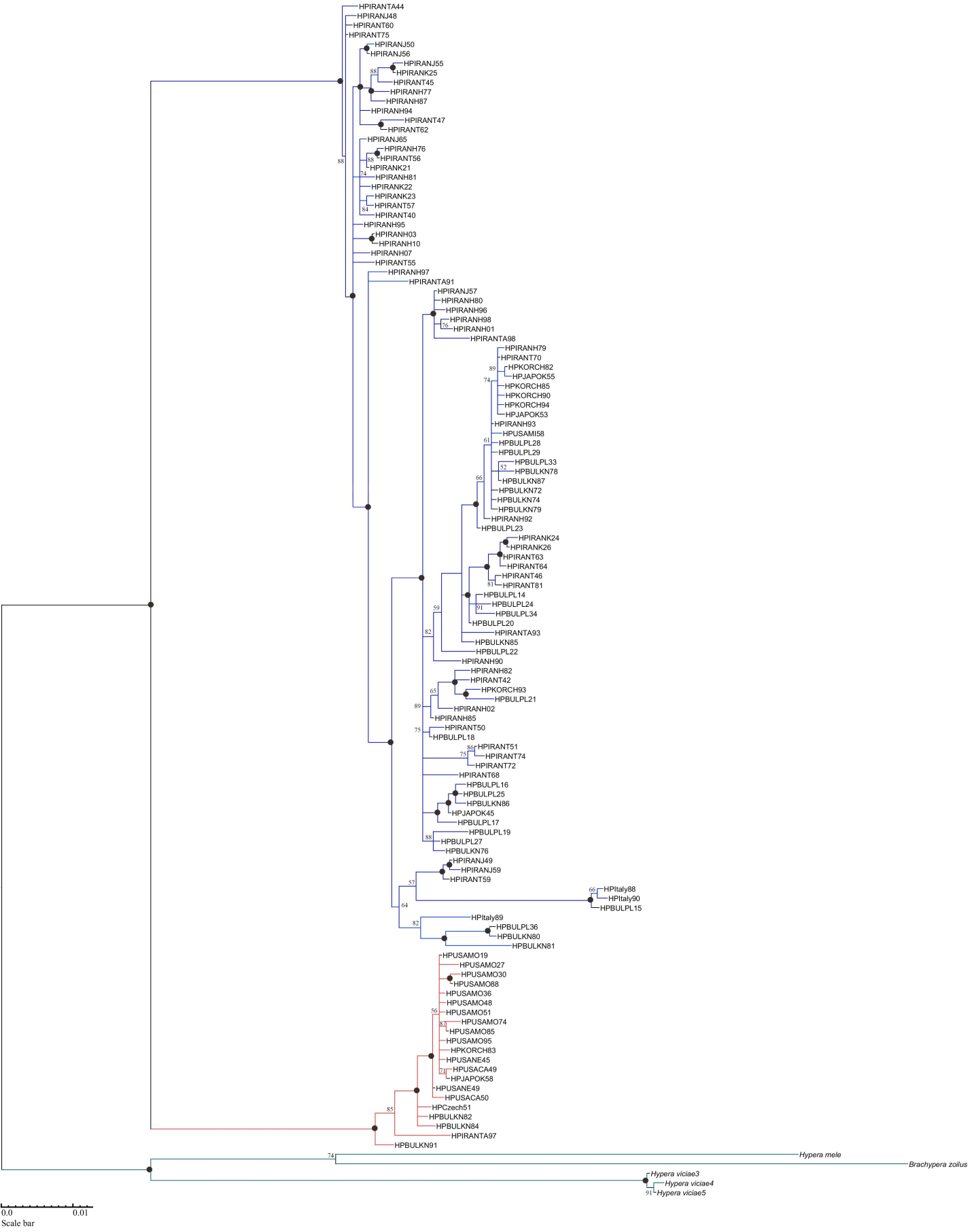


Supp. Fig. S3: Bayesian tree based on alfalfa weevil EF1a sequences. Each node number represents the posterior probability value and the black circle is the posterior probability value above 95%.


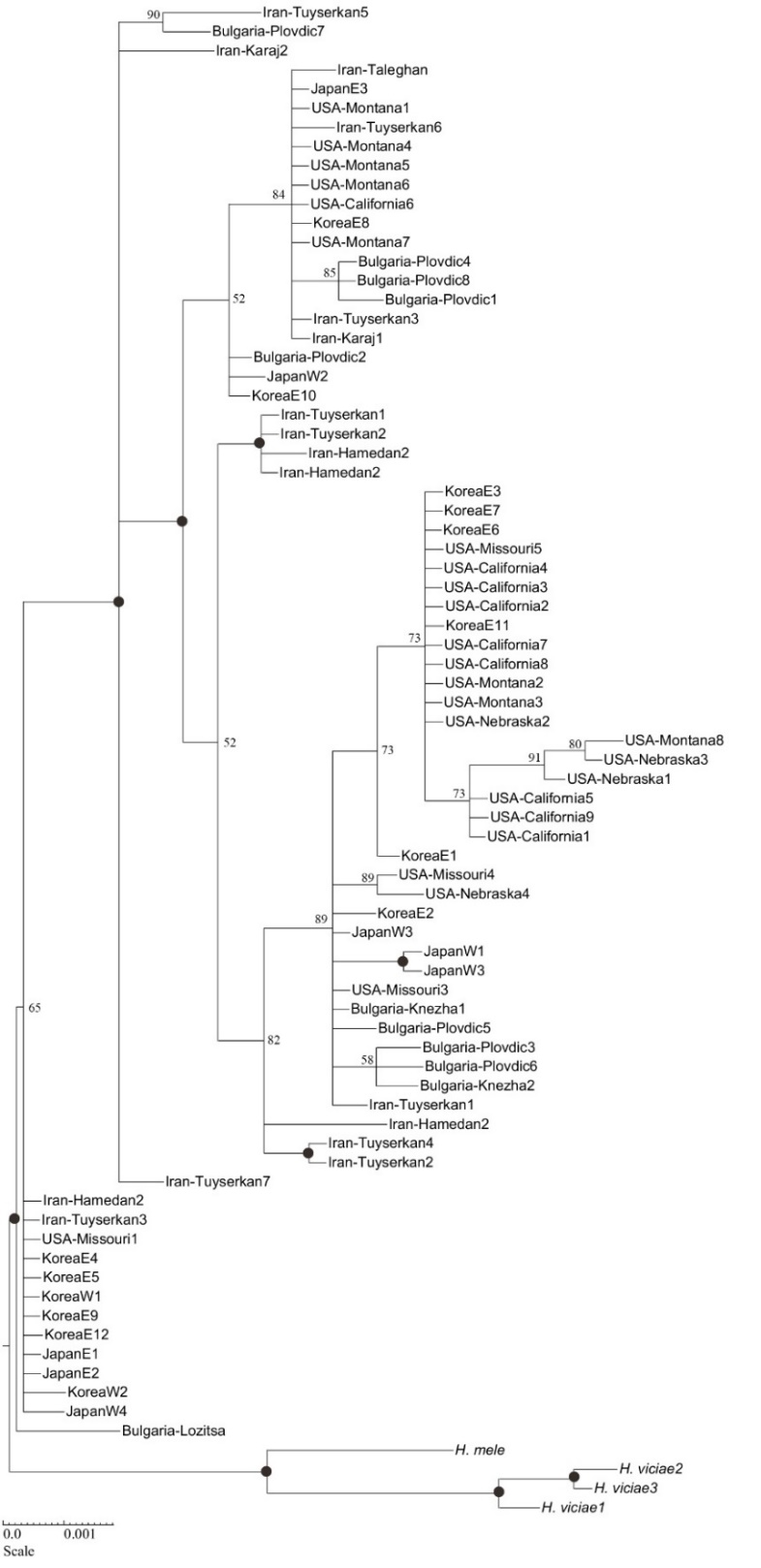


Supp. Fig. S4. Bayesian tree based on alfalfa weevil CAD sequences. Each node number represents the posterior probability value and the black circle is the posterior probability value above 95%.


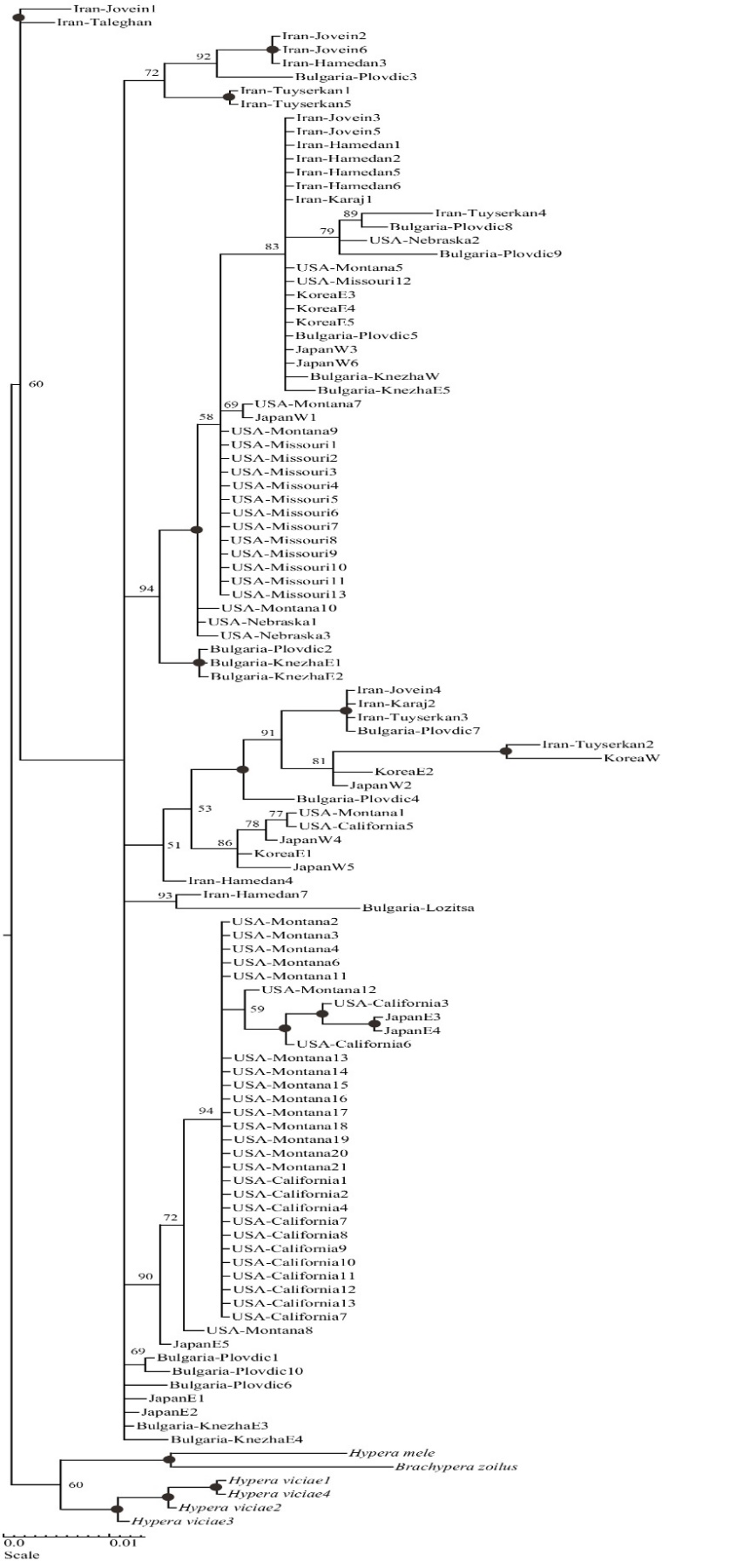


Supp. Fig. S5. Phylogenetic tree of *Wolbachia* strains based on gatB, coxA, and fbpA sequences. *w*Hypera1-3 are *H. postica* strains found in this study and other strains are selected from other arthropod’s hosts (Supp. Table S2). Each node number represents the posterior probability value and the black circle is the value above 95%. The provided ID refers to unpublished data in the MLST database ([https://pubmlst.org/*Wolbachia*/](https://pubmlst.org/Wolbachia/)). The position of *w*Hypera1-3 in Supergroup B was also confirmed by ClonalFramML approach.


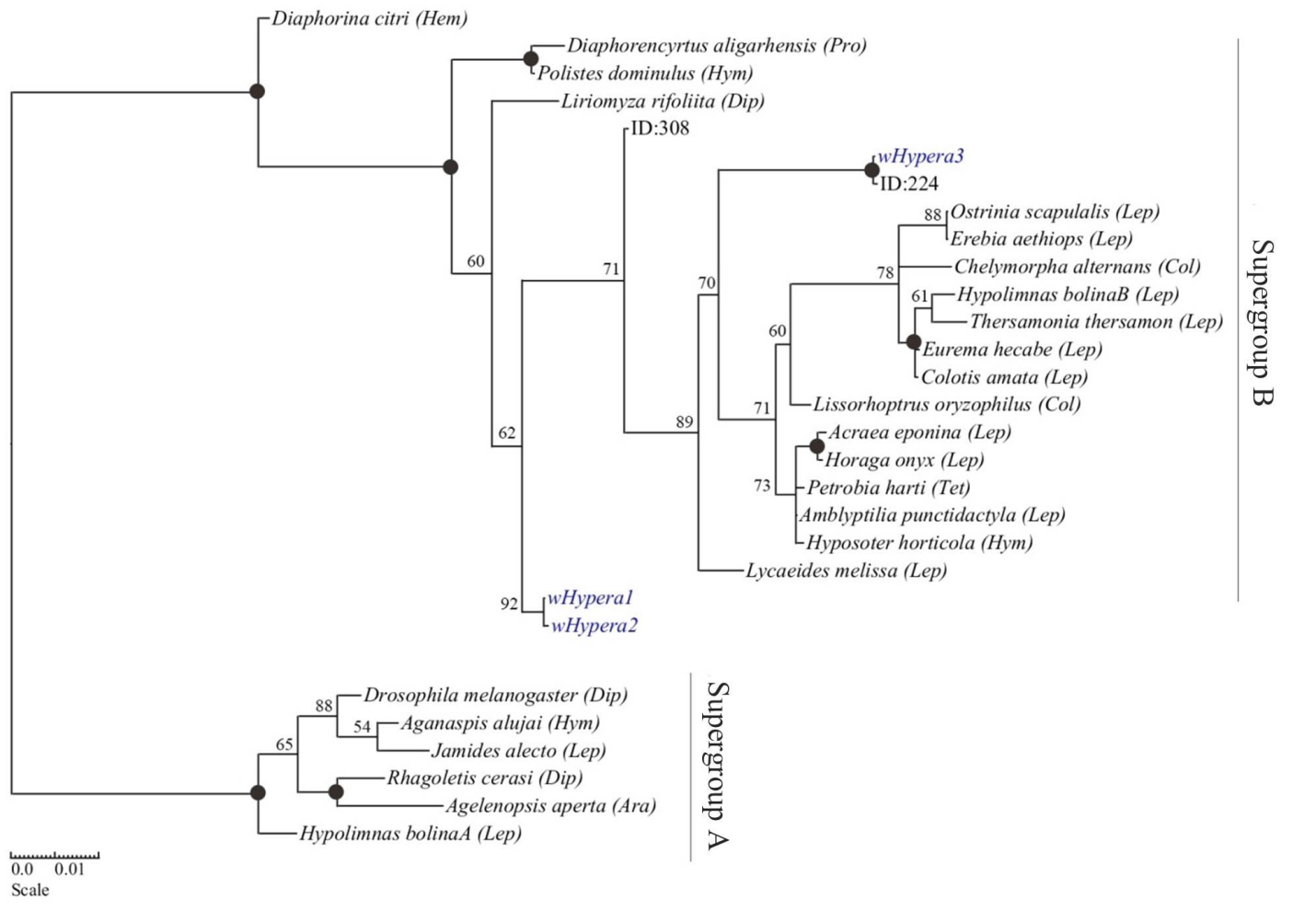

Supplement: Supplementary file 4 [file ECE3-9-9546-s004.docx]
